# Coordinated changes in the expression of Wnt pathway genes following human and rat peripheral nerve injury

**DOI:** 10.1371/journal.pone.0249748

**Published:** 2021-04-13

**Authors:** Arie C. van Vliet, Jinhui Lee, Marlijn van der Poel, Matthew R. J. Mason, Jasprina N. Noordermeer, Lee G. Fradkin, Martijn R. Tannemaat, Martijn J. A. Malessy, Joost Verhaagen, Fred De Winter

**Affiliations:** 1 Laboratory for Neuroregeneration, Netherlands Institute for Neuroscience, An Institute of the Royal Academy of Arts and Sciences, Amsterdam, The Netherlands; 2 Department of Neurosurgery, Leiden University Medical Center, Leiden, The Netherlands; 3 Department of Bioengineering, Stanford University, Stanford, CA, United States of America; 4 Department of Neurobiology, University of Massachusetts Medical School, Worcester, MA, United States of America; 5 Department of Neurology, Leiden University Medical Center, Leiden, The Netherlands; 6 Center for Neurogenomics and Cognitive Research, Neuroscience Campus Amsterdam, Vrije Universiteit Amsterdam, Amsterdam, The Netherlands; Szegedi Tudomanyegyetem, HUNGARY

## Abstract

A human neuroma-in continuity (NIC), formed following a peripheral nerve lesion, impedes functional recovery. The molecular mechanisms that underlie the formation of a NIC are poorly understood. Here we show that the expression of multiple genes of the Wnt family, including Wnt5a, is changed in NIC tissue from patients that underwent reconstructive surgery. The role of Wnt ligands in NIC pathology and nerve regeneration is of interest because Wnt ligands are implicated in tissue regeneration, fibrosis, axon repulsion and guidance. The observations in NIC prompted us to investigate the expression of Wnt ligands in the injured rat sciatic nerve and in the dorsal root ganglia (DRG). In the injured nerve, four gene clusters were identified with temporal expression profiles corresponding to particular phases of the regeneration process. In the DRG up- and down regulation of certain Wnt receptors suggests that nerve injury has an impact on the responsiveness of injured sensory neurons to Wnt ligands in the nerve. Immunohistochemistry showed that Schwann cells in the NIC and in the injured nerve are the source of Wnt5a, whereas the Wnt5a receptor Ryk is expressed by axons traversing the NIC. Taken together, these observations suggest a central role for Wnt signalling in peripheral nerve regeneration.

## Introduction

Peripheral nerve injuries can cause life-long functional disability in patients. The regenerative response of an injured peripheral nerve is dependent on a variety of parameters, including the age of the patient, the distance of the lesion to the end organs, and the severity of the injury [1]. Following injury to a human peripheral nerve often a neural scar is formed at the site of the lesion, which hampers regeneration. Occasionally, the cellular response in an injured human nerve culminates in the formation of a neuroma-in-continuity (NIC). The biological processes that underlie the formation of a NIC following injury and how the NIC inhibits functional recovery remain poorly understood. In an effort to delineate the changes in gene expression that occur in an injured human peripheral nerve, Tannemaat et al. performed a microarray analysis on NIC tissue derived from eight patients with severe brachial plexus injury [2]. 722 genes were differentially expressed in the NIC compared to the proximal nerve stump.

Interestingly, the Wnt pathway was significantly overrepresented in the set of differentially expressed genes. The Wnt gene family is of particular interest in the process of peripheral nervous system (PNS) regeneration. Canonical and non-canonical Wnt signalling pathways have been shown to play various roles in tissue repair after damage. Canonical Wnt pathway signalling is Beta-catenin dependent and acts through the regulation of gene transcription, and has widely been implicated in tissue regeneration [3–5], stem cell maintenance [6, 7], cell proliferation [8, 9] and cell differentiation [10, 11]. In addition, Wnts can act as both attractive and repulsive axon guidance molecules [12]. The non-cononical, Beta-independent, Wnt signalling pathway is mediated by calcium signalling and has been shown to regulate axon guidance during CNS development [13–15] and the inhibition of CNS axon regeneration after damage [16, 17]. Following spinal cord injury Wnt5a is expressed in the neural scar that forms at the injury site and axonal expression of the Wnt receptor Ryk mediates the repulsive effects of Wnt5a on axon regeneration. Non-canonical Wnt signalling has thus been implicated as a contributing factor in the inhibition of axon regeneration following spinal cord injury [16, 17].

Several components of the Wnt gene family have been studied in PNS development. For instance, Wnt ligands have been shown to guide the differentiation from neural crest cells towards Schwann cells [18–20]. Furthermore, Wnt3a together with R-spondin mediate Schwann cell lineage progression during PNS development and instigate the process of radial sorting [19]. Also, Wnt5a stimulates organ innervation by the sympathetic nervous system. Wnt5a knockout (KO) animals display deficits in the innervation pattern of intercostal nerves [21]. The axonal effects of Wnt5a during PNS development are mediated through an autocrine loop of the Wnt receptor Ror2 [22]. Surprisingly little is known about Wnt signalling in PNS regeneration, except for the finding that the expression of the Wnt-ligands Wnt1, Wnt3, Wnt8a and Wnt11 is upregulated and that Wnt2 and Wnt4 are downregulated in injured Dorsal Root Ganglia (DRG) neurons. In addition, it has been shown that the expression of the Wnt receptor Ryk is up-regulated in DRG neurons after a peripheral nerve injury [23, 24].

In different cellular contexts, Wnt5a has either been described as a chemo-repulsive [15–17, 25–27] or a neurotrophic factor [13, 22, 28]. It has been proposed that different Wnt receptor expression patterns are responsible for the activation of distinct intracellular signalling cascades [14, 29, 30] which mediate repulsive or neurotrophic effects. Therefore, detailed insight in which Wnt ligands, receptors, and modulators expressed under specific conditions is essential in order to understand the functional involvement of Wnt ligands in axon regeneration and tissue repair following peripheral nerve injury.

The aim of this study is to provide a comprehensive analysis of the regulation of Wnt pathway genes in the context of PNS injury and regeneration in the rat and human nerve. To this end we first verified changes in these genes in human NIC identified in a microarray study and confirmed Wnt5a up-regulation by immunohistochemistry. We then quantified the expression of Wnt gene transcripts over a range of post-lesion time points in the proximal and distal portions of crushed or transected sciatic nerves and in the dorsal root ganglia, in rats. After sciatic nerve crush injury the endoneurial tubes remain intact and most axons readily regenerate towards the muscle and skin through their original endoneurial tube. Following a sciatic nerve transection and subsequent repair both the axons as well as the endoneurial tubes are interrupted. The severed axons have to cross from the proximal to the distal nerve stump and have to find a suitable endoneurial tube before they can regrow towards and re-innervate the target tissues. As a result, regeneration of a transected nerve is more challenging than recovery of crushed nerves. Here, it was investigated whether these different injuries affect Wnt gene expression. In the injured nerve four clusters of Wnt-pathway genes with distinct temporal patterns of expression were observed. The F-Spondin1 and Wnt2 named clusters are mainly composed of Wnt ligands, including Wnt5a, Wnt5b and Wnt4, which are up-regulated as early as 3 days post-injury. A third gene cluster consists of genes that are first down-regulated and subsequently return to baseline level. This pattern of expression strongly correlates with the expression pattern of myelin basic protein (MBP), a protein component of myelin. Finally, the Axin2 cluster is characterized by a set of transcripts up-regulated from 14 days onward. In human NIC, Wnt5a was the most significantly up-regulated Wnt ligand. In the rat several Wnt ligands, including Wnt5a/b, Wnt4 and the Wnt receptor Ryk responded to nerve injury. Wnt5a/b is expressed in Schwann cells in the injured rat peripheral nerve and in the NIC.

## Material and methods

### Human material

In total, the proximal and distal nerve stump and neuroma-in-continuity of 14 patients (average age of the patients was approximately 5 months (141.3 +/- 5.4 days, Table 1) with an obstetrical brachial plexus injury were investigated in this study. All human material was collected in the Netherlands by the Neurosurgery department of Leiden University Medical Centre (Leiden, The Netherlands) during nerve reconstruction surgery and was stored at -80°C by the Pathology department of Leiden University Medical Centre (Leiden, The Netherlands). All human material was anonymized as stated in the code of conduct for responsible use of human tissue and medical research (2011) [31]. During nerve reconstructive brachial plexus surgery the NIC was identified and classified avulsion, partial avulsion, neurotmesis, intraforaminal neurotmesis, axonotmesis, or normal, based on CT myelography results, intraoperative morphological characteristics, direct nerve stimulation, and frozen-section examination [32]. NIC were determined by the following features, the spinal nerve should look normal at the intraforaminal level, should have an enlarged diameter at the juxtaforaminal level, should show epineurial fibrosis, disrupted fascicular continuity, increased consistency, and increased length of the nerve elements with concomitant distal displacement of the trunk divisions [32]. Direct electrical stimulation of the spinal nerve proximal to the lesion did not result in contraction of the associated muscle groups strong enough to induce limb movement [33]. Neuroma tissue was obtained from the superior trunk of the brachial plexus. The portion of the spinal nerve C5 or C6 directly adjacent to the neuroma was taken as proximal nerve stump tissue and served as internal control tissue. Resected neuroma, and corresponding proximal and distal stump tissue were snap-frozen within 15 minutes and stored at -80˚C. Human material was sectioned on a cryostat in 20 or 40μm sections. Sections were stored in -80˚C until further use.

**Table 1:** Matrial used for micro-array and qPCR gene expression profiling.

PATIENT	AGE	EXPERIMENTS	
**1**	127	micro-array	qPCR
**2**	130	micro-array	qPCR
**3**	169	micro-array	qPCR
**4**	148	micro-array	qPCR
**5**	108	micro-array	qPCR
**6**	138	micro-array	qPCR
**7**	116	micro-array	qPCR
**8**	146	micro-array	qPCR
**9**	151		qPCR
**10**	159		qPCR
**11**	167		qPCR
**12**	156		qPCR
**13**	110		qPCR
**14**	153		qPCR

Table lists the patient ages at the day of surgery (equals the number of days after injury) and the method of tissue analysis.

### RNA isolation and cDNA production from human tissue

Human NIC and corresponding proximal and distal stumps of 14 patients were sectioned on a cryostat 40μm sections and immediately placed in 1ml Trizol Reagent (15596018; Thermo Fisher Scientific, Waltham, MA, USA) and stored at -80˚C. Tissue was homogenised with an ultra-turrax (IKA-Labortechnik, Staufen DE). Phase separation was performed by application of Trizol solution in combination with chloroform to a phase lock gel tube (5prime, Hilden DE). The aqueous phase was transferred to an RNAse-free tube and mixed with an equal volume of 70% ethanol. Subsequently, Samples were loaded to a RNeasy Mini column (Qiagen, Valencia, CA, USA) and processed according to the RNeasy mini protocol (from step 3). The RNA concentration was determined by measuring the absorption at 260nm on a nanodrop ND-1000 (Nanodrop Technologies, Wilmington, DE, USA). The RNA quality was determined on a Bioanalyzer (Agilent technologies, Santa Clara, CA, USA) and RNA was only included when the RNA integrity number (RIN) was ≥5.5. 150ng of RNA was used for cDNA production using the Quantitect reverse transcription kit (Qiagen, Valencia, CA, USA).

### Micro-array based human gene expression analysis

Micro-array analysis is performed as described earlier [34]. Briefly, NIC and proximal stump tissue of eight patients was dissected and total RNA was isolated and analysed for concentration and quality, as described above. Micro-array analysis was performed with Agilent 44K Whole Human Genome arrays (Agilent technologies, Santa Clara, CA, USA). Sample labelling, hybridization and processing were performed according manufacturer instructions. Micro-arrays were scanned with an Agilent DNA Micro-array Scanner at 5μm resolution. Scans were quantified using Agilent feature extraction software (version 8.5.1). Raw gene expression data were imported in R statistical processing environment and analysed by LIMMA package in Bioconductor (www.bioconductor.org). A Fischer-exact test with a Bonferroni correction for multiple testing was used to determine significant differences in gene expression between NIC and proximal stump.

### qPCR based human gene expression analysis

cDNA was 20x diluted in water, and used for qPCR analysis. A SyBR green kit was used as described and the PCR procedure was performed on ABI 7900 HT (Applied Biosystems, Foster city, CA, USA). Data were analysed by 7300 Sequence detection software 1.4 (Applied Biosystems). The reference genes were B2M, YWHAZ and UBC, identified in accordance with [35].

### Animals

All animal experiments were approved by the KNAW animal ethical committee and were in accordance with European directive 2010/63/EU. Adult female Wistar rat (weight rage 180–200 gram) were purchased from Harlan (Indianapolis, IN, USA) and housed under standard conditions at a 12:12 h light/dark cycle with *ad libitum* access to water and food.

### Animal surgery

One-hundred-sixteen adult female Wistar rats received either a unilateral nerve crush or transection and co-aptation of their left sciatic nerve under hypnorm (Fentanyl; 0.08ml/100g i.m.)/dormicum (Midozolam; 0.05ml/100g s.c.) anaesthesia, in accordance with [34, 36]. Directly after the surgery and 1 day after surgery all animals received Temgesic (0.03ml/100g s.c.) as analgesic.

### Animal material for Immunohistochemistry

Fifty-eight rats were anesthetized using sodium pentobarbital and subsequently transcardially perfusioned with 4% paraformaldehyde (PFA) in 0.1M PBS, pH 7.4, at 3, 5, 14, 28, 42 and 90 days after injury. The sciatic nerves and corresponding L4 and L5 DRG’s were dissected and post-fixed in 4% PFA in 0.1M PBS, pH 7.4, O/N at 4˚C, after which tissue was cryoprotected in 25% sucrose in 0.1M PBS pH 7.4 O/N at 4˚C. All tissue was snap-frozen on dry ice and embedded in Tissue-Tek (Sakura, Alphen aan den Rijn, Netherlands) and kept at -20˚C until 20μm sections were made on a cryostat.

### Immunohistochemistry

Sections were immersion-postfixed in 4% PFA in 0.1M PBS, pH 7.4 for 15 minutes at R.T. Immunohistochemistry (IHC) was performed in Tris-buffered saline (TBS), pH 7.4, containing 0.2% Triton X100 and 5% fetal calf serum. IHC was performed with the following antibodies: mouse anti-neurofilament (1:1000; 2H3 ascites; Developmental Studies Hybridoma Bank, University of Iowa, IA, USA), rabbit anti-S100, rabbit anti-Wnt5a (1:100; for human tissue; #2530; Cell signalling, Beverly, MA, USA) goat anti-Wnt5a (1:100; for human tissue; AF645; R&D systems, Minneapolis, MN, USA), rabbit anti-Wnt5a (1:100; for rat tissue; Abcam, Cambridge, UK, USA), rabbit anti-Ryk (1:500; a gift from Dr. Yimin Zou), rabbit anti-calnexin (1:200; ab22595; Abcam, Cambridge, UK). Stained sections were imaged on a Leica SP5 confocal microscope. Quantification of the immunostaining was done in ImageJ using a fixed threshold for each marker for all sections. Photo panels were put together using ImageJ and Adobe Photoshop.

### RNA isolation and cDNA production from rat tissue

Fifty-eight rats were sacrificed by decapitation after CO2/O2 sedation at 3, 5, 14, 28, 42 and 90 days after injury. Three 6mm sciatic nerve segments (one proximal to, one around the injury site and one distal to the injury site) and corresponding L4 and L5 DRG’s were dissected and immediately placed in 1ml Trizol Reagent (Ambion; 15596018) and stored at -80˚C. RNA isolation was performed by first applying Trizol to a phase lock gel (5prime, Hilden DE), followed by isolation on RNAeasy mini columns (Qiagen, Valencia, CA, USA). RNA concentration was determined by 260nm absorption on a nanodrop 1000 spectrophotometer (Thermo scientific; Waltham, MA, USA) 150ng of RNA was used for cDNA production using the Quantitect reverse transcription kit (Qiagen, Valencia, CA, USA).

### qPCR on rat cDNA

cDNA was 20x diluted in mQ, and employed for qPCR. SyBR green kit was used as described and PCR procedure was performed on ABI 7900 HT (Applied Biosystems, Foster city, CA, USA) data were analysed by 7300 Sequence detection software 1.4 (Applied Biosystems, Foster city, CA, USA). The reference genes were GAPDH, NSE and β-actin in accordance with [37]. qPCR data is shown in heatmaps generated by hierarchical clustering. The data are plotted as 2Log values compared to a healthy uninjured nerve or L5 DRG. qPCR statistical analysis was performed by two-way-ANOVA in R statistical processing environment (multicomp package), with multiple testing correction by Benjamini Hochberg algorithm. Genes that exhibited significant regulation (p<0.05) over the entire timeline were selected for Dunnets post-hoc analysis and compared to uninjured nerve or DRG to identify significant timepoints.

### Primers

All primers were designed on the Primer3 NCBI website with melting temperature ~60˚C. A complete list of all primer sequences is provided in as a S1 Table (Primer list).

## Results

### Specific WNT genes are differentially expressed in the human NIC

**Fig 1:**
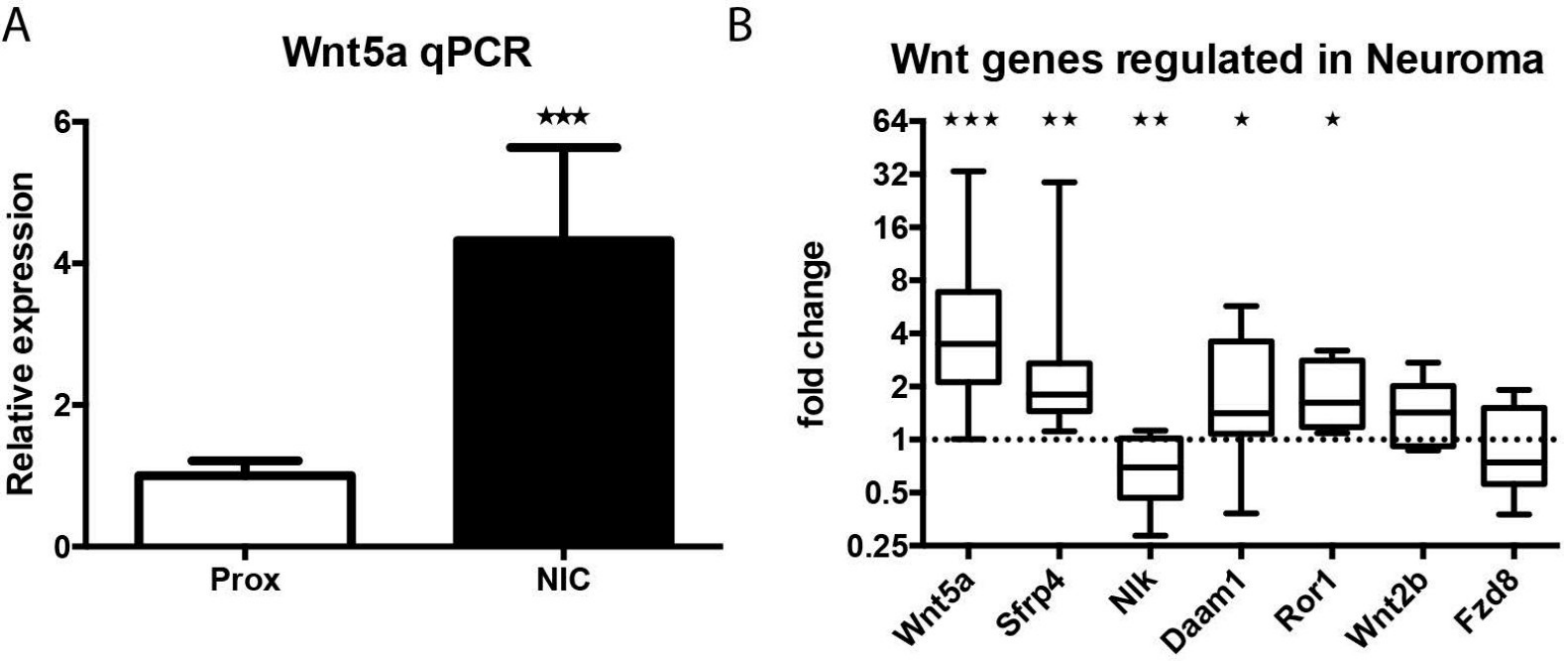
Specific Wnt pathway genes are differentially expressed in human NIC. *A*, qPCR analysis shows that 5 months after injury human NIC tissue has increased levels of Wnt5a mRNA compared to the proximal stump. *B*, in addition to Wnt5a (p<0.001), qPCR analysis shows that extracellular Wnt modulator Sfrp4, Wnt pathway member Daam1 and the Wnt receptor Ror1 are upregulated. Wnt pathway member Nlk is down regulated. Wnt receptor Fzd8 and Wnt ligand Wnt2 are not differently expressed between the NIC and the proximal stump (* p<0.05, ** p<0.01, *** p<0.001).

To gain insight in the gene expression changes that occur in human neuroma-in-continuity (NIC) a micro-array analysis was performed on NIC tissue of eight patients after neonatal brachial plexus injury (NBPI). A total of 722 genes were differentially expressed between the NIC and the proximal nerve stump [2]. We chose the nerve stump immediately proximal to the NIC as control tissue as non-injured nerve tissue from the contralateral side is not available in human patients. Sections of the proximal nerve adjacent of the NIC were examined during the reconstructive surgery and the proximal stump was considered a healthy nerve as 80% or more of transversal section surface had a normal organized nerve structure and myelin. The complete gene expression data set will be published elsewhere. In this study we focus on changes in gene expression of the Wnt pathway. The expression of seven members of the Wnt pathway was significantly changed, namely, Wnt5a, Wnt2b, Sfrp4, Fzd8, Ror1, Daam1, Nlk (Table 2). A Fisher exact test demonstrates that the Wnt pathway was significantly overrepresented in the set of significantly regulated genes (p<0.05). qPCR validation of the expression of these Wnt genes was performed on an extended patient group, which included the eight original patients used for the microarray study and six new patients (Fig 1A and 1B). qPCR analysis of human NIC tissue corroborated that Wnt5a, Sfrp4, Ror1, Daam1 and NLK are differentially expressed. Using qPCR, no significant differences were observed for Wnt2b and Fzd8. Taken together, we show that a Wnt ligand (Wnt5a), a Wnt receptor (Ror1), and two intra-cellular downstream Wnt signalling factors (Daam1 and NLK) are differentially expressed in NIC.

**Table 2:** Wnt pathway genes are differentially expressed in human NIC.

Gene	Fold Change	p-Value
**Wnt5a**	3.99	0.044
**Sfrp4**	2.79	0.046
**Wnt2b**	2.01	0.023
**Daam1**	1.92	0.026
**Ror1**	1.55	0.043
**Fzd8**	-1.51	0.018
**Nlk**	-1.53	0.034

Micro-array analysis of human NIC material shows the regulation of several members of the Wnt pathway.

### Wnt5a is expressed by Schwann cells of the NIC

**Fig 2:**
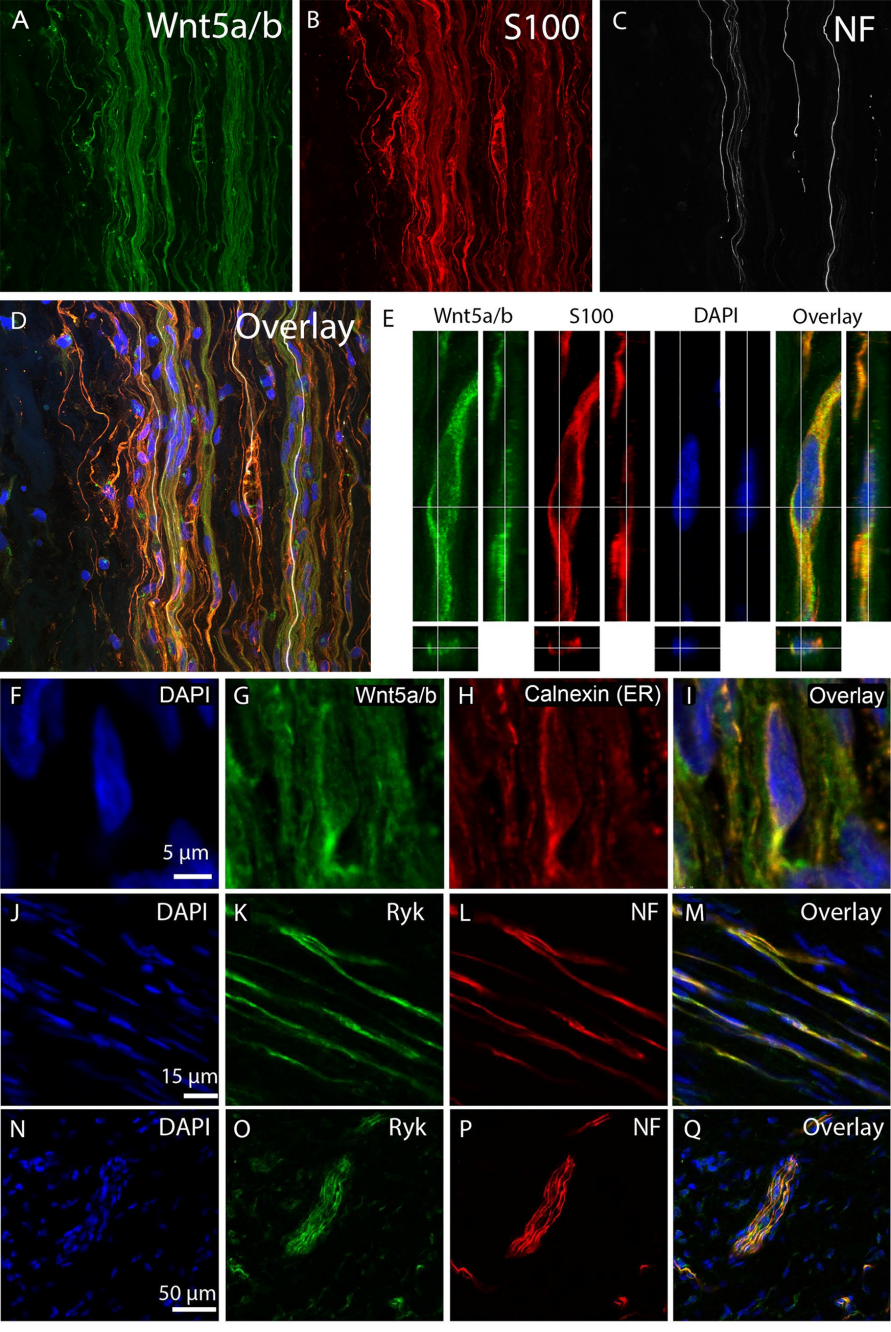
Wnt5a/b is expressed by Schwann cells in NIC. A-E, Immunohistochemistry for Wnt5a/b on human NIC tissue sections. Wnt5a/b (A, green) is expressed by S100 (B, red) positive Schwann cells in the NIC. Neurofilament (NF) positive axons (C, gray) that traverse the NIC are closely associated with the Schwann cells (D, overlay (Including nuclear marker DAPI, blue)). E, High magnification with orthogonal view of an immunohistological staining for Wnt5a/b (green), S100 (red) and DAPI (blue) shows the presence of Wnt5a/b protein in a S100 positive Schwann cell. F-I, Strong Wnt5a/b (G, green) expression is visible in intracellular perinuclear structures (blue in F). Co-staining with the ER-marker calnexin (H, red) reveals that Wnt5a/b is expressed in the ER (I, overlay). J-M, N-Q, Cellular localisation of Ryk in NIC. Co-staining for DAPI, Ryk and Neurofilament reveals that Ryk expression (green in K, O) is observed on individual neurofilament positive axons (L, red) and in axon bundles (P, green) that traverse the human NIC (M, Q, overlay). Panel N to Q also show that Ryk is also weakly expressed around the nuclei (blue in N) of cells outside axon bundles in the NIC (Q, overlay).

Wnt5a was selected as a promising gene for further study because of its role in axon outgrowth [13–15], neurogenesis [38, 39], differentiation [40–42], proliferation [43–45] and fibrosis [46]. All these processes are of considerable interest in the context of the formation of a NIC. To our knowledge all commercially available Wnt5a antibodies recognize both Wnt5a and Wnt5b. We used Wnt5a/b antibodies to investigate the cellular localisation of Wnt5a/b in NIC by a triple immuno-histochemical analysis for Wnt5a/b, a Schwann cell marker (S100) and Neurofilament (NF). Cell nuclei were stained with DAPI (Fig 2). Wnt5a/b was localized to intra-fascicular cells (Fig 2A) that were S100 positive (Fig 2B, overlay in 2D). In the NIC is 82.1% of the Wnt5a/b positive area also marked by S100 (n = 6, SEM ± 2.7), suggesting that most of the Wnt5a/b is expressed by Schwann cells. Axons (NF) that traverse the NIC are often found in close association with these Wnt5a/b positive Schwann cells (Fig 2C, overlay in 2E). Wnt5a/b is distributed in small puncta throughout cytosol of S100 positive Schwann cells (example cell in Fig 2E). Interestingly, in many cells also a distinct peri-nuclear staining pattern is visible for Wnt5a/b (Fig 2G). Wnt5a/b co-localized with the ER marker calnexin (Fig 2H, overlay in 2I). This shows that intracellular Wnt5a/b is at least partly localised to the ER. In addition, there is a more diffuse extracellular staining for Wnt5a/b present in the NIC (2G), indicating that Schwann cells may secrete Wnt5a/b.

### Ryk is expressed by neurites that populate the human neuroma-in-continuity

The Wnt5a receptor Ryk mediates the chemorepulsive effects of Wnt5a in the developing CNS and following injury to the spinal cord [15–17]. To investigate whether the nerve fibres in the NIC express Ryk, an IHC double staining was performed for Ryk and NF (Fig 2J–2M). Ryk and NF exhibit significant co-localisation (Fig 2J–2Q), which indicates that this Wnt5a receptor is expressed in axons in a NIC. In addition to the distinct axonal Ryk expression, a relatively low Ryk signal is observed in many other cells in the NIC (Fig 2N–2Q).

### Expression analysis of Wnt signalling pathway genes following peripheral nerve injury in the rat

The observations on the expression and localization of Wnt5a mRNA, Wnt5a/b protein and Ryk in human neuroma tissue prompted us to study the expression of Wnt ligands and their receptors in the injured rat peripheral nerve. We employed two rat PNS injury models, sciatic nerve crush and sciatic nerve transection. After sciatic nerve crush injury the endoneurial tubes remain intact and most axons readily regenerate towards the muscle and skin through their original endoneurial tube. Following a sciatic nerve transection injury and subsequent repair both the axons as well as the endoneurial tubes are interrupted. The severed axons have to cross from the proximal to the distal stump and have to find a suitable endoneurial tube before they can regrow towards and re-innervate the target tissues. As a result, regeneration of a transected nerve is more challenging than recovery of crushed nerves. In both injury paradigms we quantified transcript expression over a range of time points chosen to correspond to relatively well-defined aspects of the regeneration process, including the initiation of axonal regeneration, Schwann de-differentiation and proliferation (3 and 5d post injury (p.i.)), axon extension and re-innervation (14, 28d p.i.) and re-myelinisation, maturation and functional recovery (28, 42, 90d p.i.). In both injury paradigms we analysed a nerve portion proximal from the injury site (prox), a nerve portion that includes the injury (inj) and a portion distal to the injury (dist).

An extensive analysis of the expression of the genes that constitute the Wnt signalling pathway, including Wnt ligands, Wnt receptors, Wnt modulators and downstream members of the Wnt pathway, revealed changes in gene expression at all levels of the Wnt pathway. The expression profiles of the Wnt genes were compared to expression of markers of immature and de-differentiated (F-Spondin1 [47–49]) and myelinating (MBP [50]) Schwann cells. Hierarchical gene clustering was performed in combination with heat map representation to show patterns of temporal co-regulation of genes. Gene clusters are named after each of the two marker genes or after a gene that represents the expression profile of the gene cluster. The expression profiles are compared to the gene expression levels of an uninjured control nerve. The fold changes and SEM are provided in the S2–S4 Tables.

**Fig 3:**
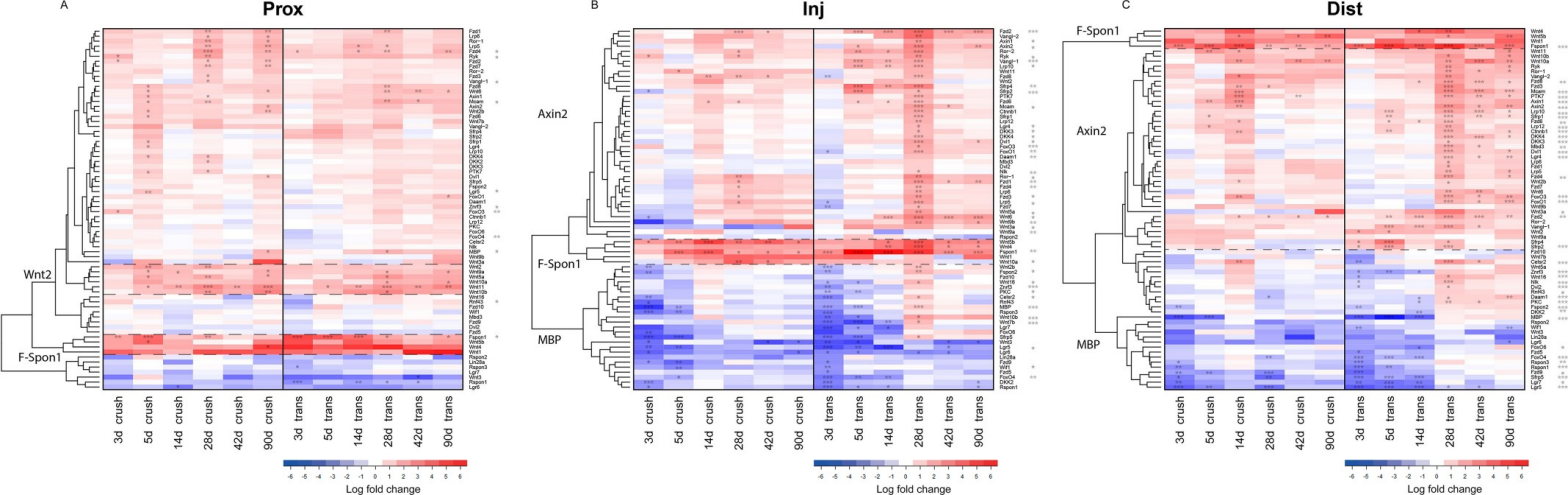
Changes in the expression of Wnt pathway genes after peripheral nerve injury in the rat. The expression of 72 Wnt pathway genes in crushed (left half of the figure) and transected (right half of the figure) rat nerve (3 to 90 days post injury) was analysed using qPCR. Data is presented as clustered heat maps for the proximal stump (A), injury site (B) and distal stump (C). Red indicates up- and blue down-regulation of mRNA expression of the analysed genes compared to uninjured control nerve. *A*, The proximal stump displays two clusters of co- regulated genes, the Wnt2 and F-Spondin cluster. *B*, *C*, the injury site (B) and distal nerve stump (C) exhibit three clusters of genes, the F-Spondin cluster, the axin-1 cluster and the MBP cluster. All corresponding fold changes and SEM are provided in S2–S4 Tables. Asterisks indicate significant timepoints, asterisks behind gene names indicate significant effects of injury on the gene expression profile (* p<0.05, ** p<0.01, *** p<0.001).

The temporal expression patterns of the Wnt pathway genes appear to follow similar trends following nerve crush and transection, although it is apparent that the transected nerve demonstrates a more profound regulation of Wnt pathway genes (Fig 3A–3C). Two-way-ANOVA analysis demonstrates that there are significant differences in the degree of Wnt pathway regulation between the two injury paradigms (crush and transection). In the proximal stump 11 of 72 tested genes are significantly differentially regulated between injury paradigms, but this does not include any of the Wnt ligands. The injury site demonstrates a higher level of differential regulation between injury paradigms, with 40 of 72 significant genes, these include 9 of the 16 Wnt ligands. Finally, the distal nerve has 41 of 72 genes significantly differentially regulated between injury paradigms, with 2 of the 16 Wnt ligands. All significantly differentially regulated genes are indicated with asterisks next to the gene name (Fig 3A–3C).

### Wnt family genes expression clusters in four distinct temporal profiles after peripheral nerve injury

**Fig 4:**
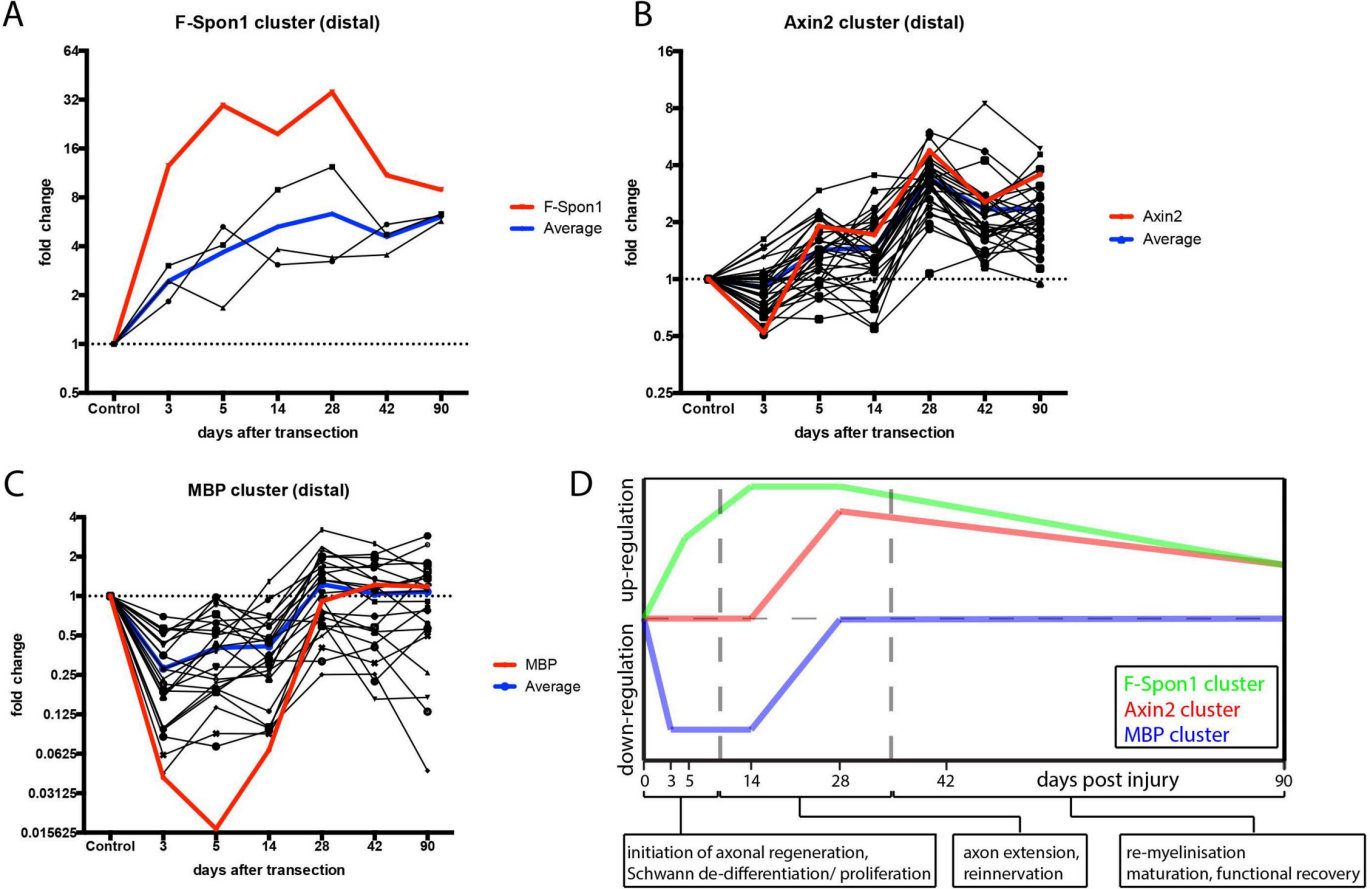
Wnt pathway gene clusters in the distal stump of the transected sciatic nerve. The temporal expression profiles of individual genes in the F-Spondin1, Axin2 and MBP clusters in the distal stump were plotted to visualize the coordinated nature of the expression of Wnt-pathway genes after nerve transection. Black lines represent individual genes, blue lines indicate average regulation and red lines indicate regulation of the marker gene (F-spon1, MBP) or the Wnt2b or Axin2 gene. *A*, The F-Spondin cluster contains genes which are up- regulated from early time points onward (3d p.i.) and remains up-regulated up to late (90d p.i.) timepoints. *B*, The genes in the Axin2 cluster exhibit an up- regulation at later time points (14–28d p.i.) and remain up-regulated up to 42 to 90d p.i. *C*, The MBP cluster consist of genes that are down-regulated at early timepoints (3 - 14d p.i.) and return to baseline around 28d p.i. *D*, Schematic representation of the F-Spondin1 (green), Axin2 (red) and MBP (blue) clusters with an indication of their correspondence to post-injury repair events in the peripheral nerve as indicated at the X-axis of the graph suggesting that these genes may have a role in these processes.

All three nerve portions exhibit a gene cluster of up-regulated genes which includes Wnt4, Wnt5b, Wnt1 and F-Spon1. We refer to this cluster as the ‘F-Spon1 cluster’. This cluster consists of genes that are up-regulated as early as 3 days after the lesion and up-regulation persists during late post-lesion time points (42-90d p.i.). This cluster is especially pronounced in the injury containing and distal nerve portions (Figs 3A–3C and 4A).

In the proximal nerve portion a second cluster of up-regulated Wnt ligands named the ‘Wnt2 cluster’ was identified. This cluster consists of genes up-regulated after 5d p.i. that remain up-regulated until 90d p.i. and consists of the Wnt ligands Wnt2, -5a, -9a, -10a, -10b and Wnt11 (Fig 3A).

The injury site and distal nerve portion both exhibit a large cluster of genes that are not regulated at early time points but are up-regulated from 14d in crush and 28d in transection injury. This cluster is referred to as the ‘Axin2 cluster’. Finally, a fourth cluster of genes was called the ‘MBP cluster’ because the temporal expression profiles of genes in this cluster followed that of MBP, exhibiting early down regulation of genes and normalisation of expression between 14 and 28 days after injury (Figs 3B, 3C, 4B and 4C). The “Axin2 cluster” consists of 40 genes, including Fzd2, Vangl1, Axin1, Axin2, Ror2, Ryk, Vangl2, Lrp10, Wnt11, Fzd8, Wnt2, Sfrp2, Sfrp4, Ptk7, Fzd6, Mcam, Sfrp1, Lrp12, Lgr4, DKK3, DKK4, Dvl1, Foxo3, Foxo1, Mdb3, Ror1, Fzd4, Fzd1, Lrp6, Fzd3, Lrp5, Fzd7, Wnt6, Wnt9b, Wnt3a, Wnt9a and B-catenin (Figs 3B, 3C and 4B). The “MBP-cluster” contains 25 Wnt-genes (including Wnt16, Fzd5, Fzd9, Sfrp5 Spon2, Rspon1, Rspon3, Wif1, Lgr5, Lgr6, Lgr7, Foxo4, Foxo6; Figs 3B, 3C and 4C).

Taken together, in the rat Wnt signalling pathway genes display coordinated changes in expression after PNS injury (summarized in Fig 4D). Genes in the F-Spon1 cluster (including Wnt4 and Wnt5b) and Wnt2 cluster (including Wnt5a) are significantly up-regulated during the early and intermediate stages of the regeneration process, suggesting that these Wnt-ligands may have a role in axon regeneration and/or Schwann cell de-differentiation (Fig 4A and 4D). Genes in the “Axin2 cluster” display changes in expression during later stages of the regeneration process (Fig 4B and 4D). These genes may therefore have roles in Schwann cell differentiation, remyelination of regenerated axons or other processes in the nerve that are important for the recovery of function of the injured nerve, e.g. target cell re-innervation. Finally, the genes in the “MBP-cluster” are down-regulated in the early stages of regeneration and return to normal levels during the later stages of regeneration (Fig 4C and 4D). Therefore, these genes may have roles in the formation and/or maintenance of myelin and the post-lesion maturation of Schwann cells.

### IHC analysis of Wnt5a/b in experimental peripheral nerve injury

**Fig 5:**
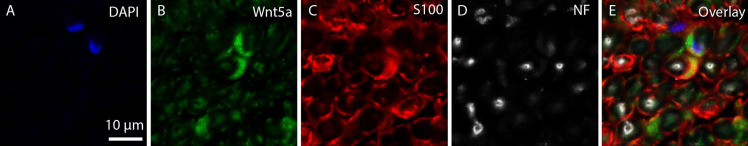
Wnt5a/b is expressed by Schwann cells in the injured rat nerve. *A-E*, Triple label Immunohistochemistry for Wnt5a/b, S100 and NF on transversal sections of rat peripheral nerve 28d after sciatic nerve crush. Wnt5a/b (B, green) is predominantly expressed in S100 positive Schwann cells (C, red) that enwrap neurofilament positive axons (D, white) in the injured rat nerve (E, overlay of panels A, B, C and D). Scale bar is 10μm.

The Wnt5b transcript is up-regulated at the injury site and in the distal nerve stump of the injured rat peripheral nerve. Wnt5a is significantly up-regulated at 28d p.i. at the injury site in the transected nerve. The cellular location of Wnt5a and Wnt5b (Wnt5a/b) was investigated by IHC. This analysis was performed on rat 28d post crush sciatic nerve (Fig 5A–5D). The antibody employed for human tissue was not functional on rat tissue, therefore another antibody (Abcam; ab72583) was used. Based on the peptide sequence it was raised to, this antibody recognizes both Wnt5a and Wnt5b. It is therefore not possible to discriminate between the expression of Wnt5b and Wnt5a protein in the nerve. Nerve sections were stained for DAPI (A), Wnt5a/b (B), the Schwann cell marker S100 (C) and NF (D). Schwann cells express Wnt5a/b, as indicated by co-expression of Wnt5a/b and S100. This is in line with the observation in NIC where Wnt5a/b is also predominantly expressed in Schwann cells. The distinct peri-nuclear staining for Wnt5a/b observed in Schwann cells in NIC tissue is not observed in rat Schwann cells.

**Fig 6:**
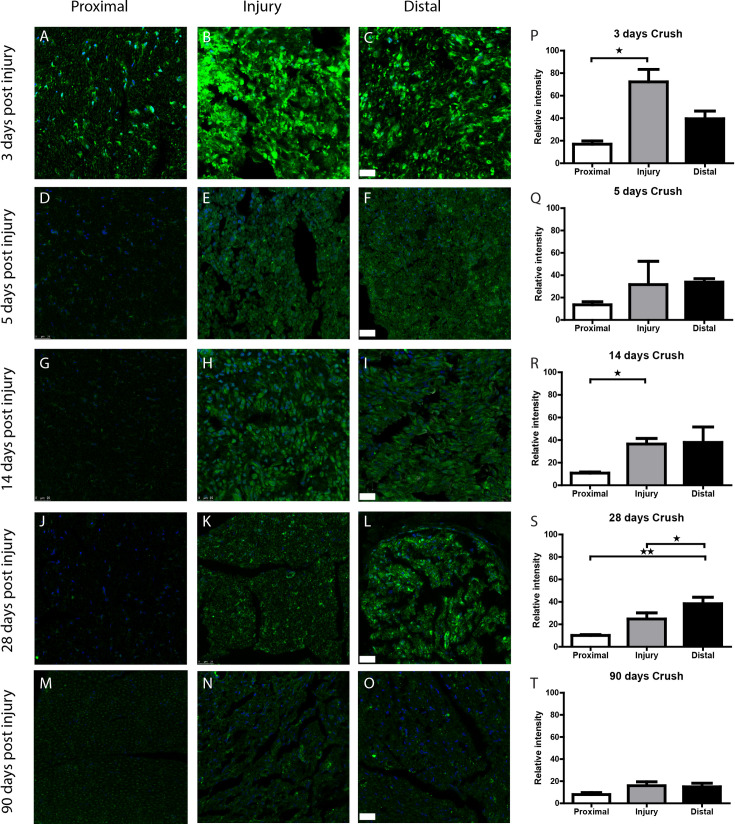
Wnt5a/b protein expression is up-regulated in the crushed rat nerve. *A-O*, Immunohistochemical staining of Wnt5a/b (green) and nuclear staining with DAPI (blue) on transversal sections through the proximal stump (left column), injury site (middle column) and distal stump (right column) of the rat peripheral nerve at 3 to 90 days after nerve crush. Wnt5a/b expression is low in the proximal stump at all time points (A, D, G, J, M). The injury site shows very high Wnt5a/b staining at 3 days (B) and this gradually diminishes in staining intensity over time from 5 (E), 14 (H), to 28d p.i. (K) and returns to baseline at 90d p.i. (N). In the distal stump Wnt5a/b expression is increased at 3 (C), 5 (F), 14 (I) and 28d p.i. (L) and has returned to baseline at 90d p.i. (O). Quantification of Wnt5a/b signal in the proximal nerve stump, injury site and distal nerve stump is shown in the graphs in panels *P to T*. (* p = <0.05, ** p = <0.01, error bars represent SEM). Scale bar is 25μm.

**Fig 7:**
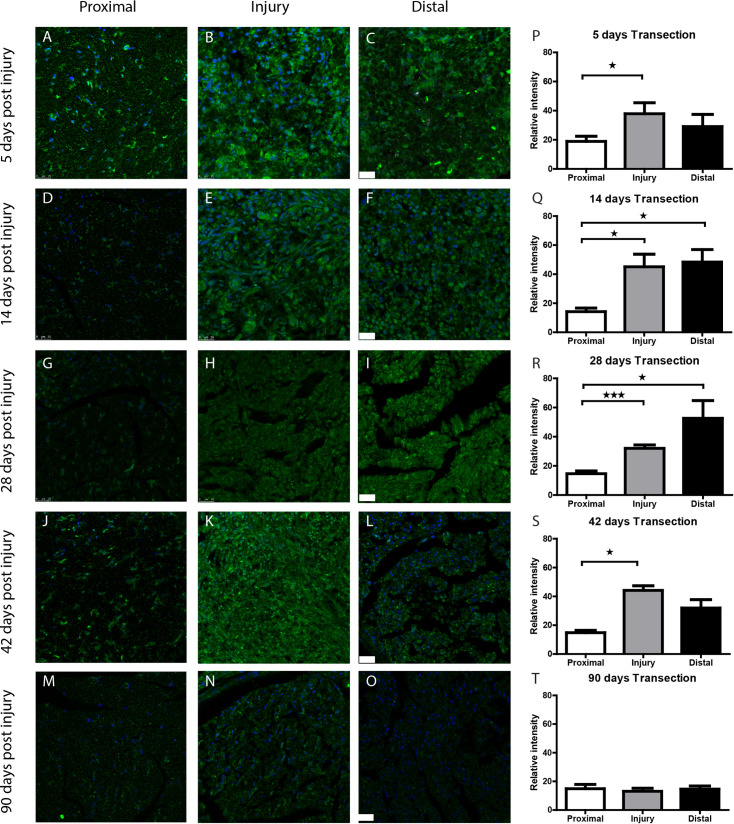
Wnt5a/b protein expression is up-regulated in the transected rat nerve. *A-O*, Immunohistochemical staining of Wnt5a/b (green) and nuclear staining with DAPI (blue) on transversal sections through the proximal stump (left column), injury site (middle column) and distal stump (right column) of the rat peripheral nerve at 3 to 90 days after nerve transection. Wnt5a/b expression is low in the proximal stump at all time points (A, D, G, J, M). The transection- coaptation site shows high Wnt5a/b staining at 3 days (B) gradually diminishing in staining intensity over time from 5 (E), 14 (H), to 28d p.i. (K) and returns to baseline at 90d p.i. (N). In the distal stump Wnt5a/b expression is increased at 3 (C), 5 (F), 14 (I) and 28d p.i. (L) and has returned to baseline at 90d p.i. (O). Quantification of Wnt5a/b signal in the proximal stump, injury site and distal nerve stump is shown in the graphs in panels *P to T*. (* p<0.05, ** p<0.01, *** p<0.001, error bars represent SEM). Scale bar is 25μm.

To quantify Wnt5a/b protein expression levels we employed the same timeline as for our mRNA expression analysis in sciatic nerve tissue. Both crushed (Fig 6) and transected (Fig 7) nerves were analysed immunohistochemically. In the proximal portion of an injured nerve, relatively low levels of Wnt5a/b are detected in Schwann cells (Figs 6 and 7). In the injury site and distal to the injury Wnt5a/b is up-regulated in Schwann cells and the up-regulation persists for at least 42 days. After 90 days, the expression of Wnt5a/b had returned to baseline. At early time-points (3-5d p.i.; Figs 6A–6C and 7A–7C), a small population of cells with intense staining of Wnt5a/b are noticeable. Considering their distribution and round shape these cells are possibly macrophages. Macrophages are known to express Wnt5a [51] and are recruited to an injured peripheral nerve at these post-lesion timepoints [52].

### Wnt receptors are regulated in DRGs after PNS injury

**Fig 8:**
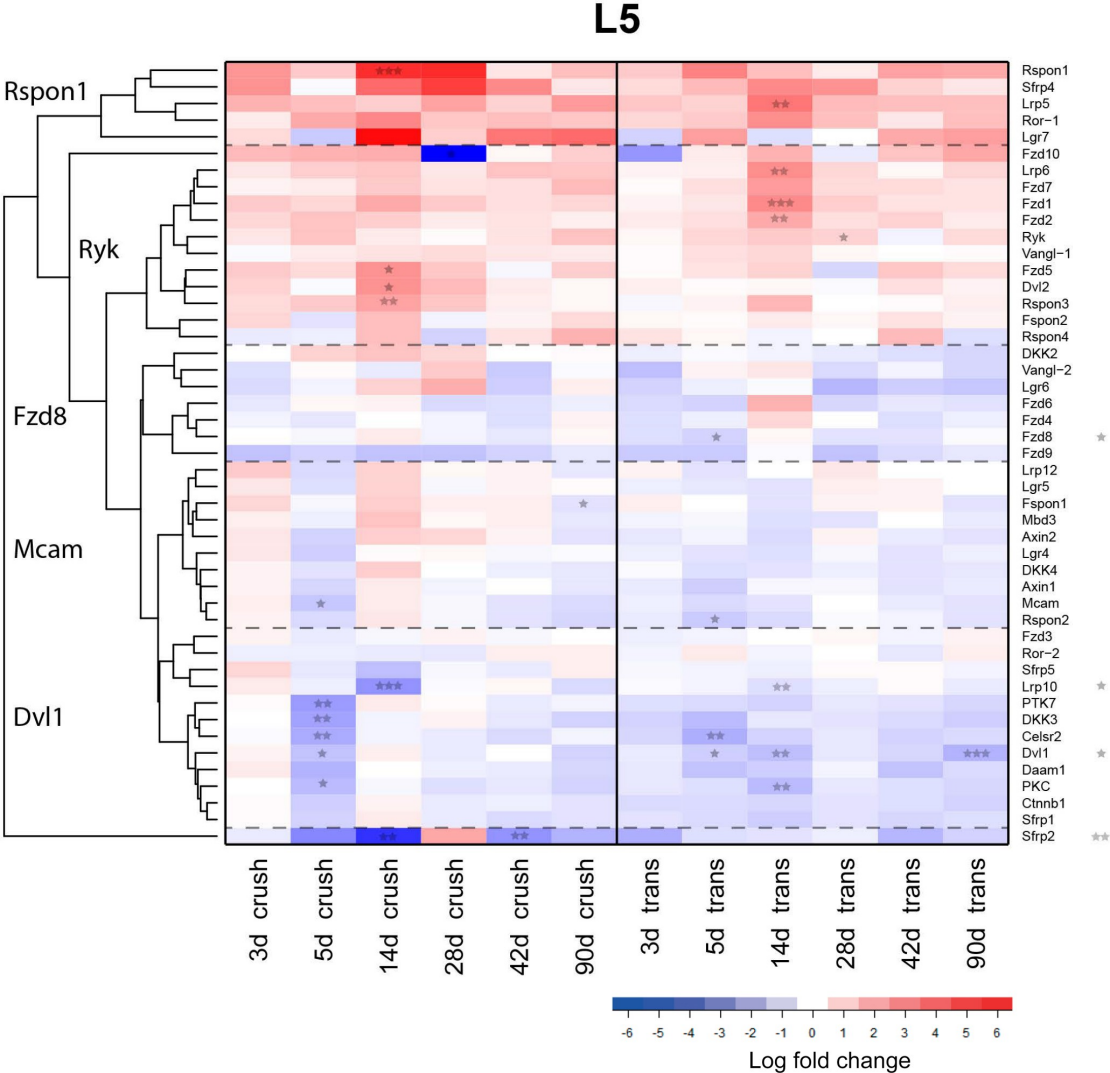
Differential expression of Wnt pathway genes in L5 DRG following sciatic nerve crush or sciatic nerve transection. Wnt-pathway gene expression was analysed in L5 DRG following sciatic nerve crush (left half) or transection (right half). In the heatmap red indicates up-regulation and blue down-regulation compared to control DRG. Five gene clusters, named the R-spondin1, Ryk, Fzd8, Mcam and Dvl1 cluster, were observed. The Ryk cluster contains 7 Wnt receptors, which mostly peak in expression at 14d p.i. All fold changes and SEM are provided in S5 Table. Significant timepoints are indicated by asterisks. Asterisks behind gene names indicate significant effects of injury on the gene expression profile (* p<0.05, ** p<0.01, *** p<0.001).

The injury-induced expression of several Wnt ligands in the sciatic nerve may have a role in functioning of the various cells present in the injured nerve (Schwann cells, fibroblasts, blood vessel cells, immune cells) and/or may have an impact on the regenerating axons themselves. To investigate the latter possibility the expression of Wnt receptors was analysed by qPCR in the L5 DRG. These DRGs contain the cell bodies of the sensory neurons of the sciatic nerve and the supporting satellite cells. Hierarchical gene clustering was performed in combination with heat map representation. All gene clusters are named after a gene that represents the expression profile of that gene cluster. The expression profiles are compared to the gene expression levels of an uninjured L5 DRG. The fold changes and SEM are provided in the S5 Table. Two-way-ANOVA analysis identified four significantly regulated genes between the crush and transection injury paradigms, namely: Fzd8, Dvl1, Lrp10 and Sfrp2 (indicated by asterisks next to the gene name (Fig 8)).

Five clusters could be distinguished. A cluster of highly up-regulated transcripts called the ‘Rspon1 cluster’ exhibits a peak of regulation at 14 to 28d p.i. and consists of 5 genes, namely: Rspon1, Sfrp4, Lrp5, Ror1 and Lgr7.

The subsequent cluster of up-regulated genes called the ‘Ryk cluster’ displays a peak in regulation at 14d post-injury. The Ryk cluster consists of 12 genes, predominantly Wnt receptors, including Fzd1, Fzd2, Fzd5, Fzd7, Ryk, Lrp6, Vangl1, Dvl2, Rspon3, Spon2 and Rspon4.

The next two clusters demonstrate a modest degree of down-regulation after injury. The Fzd8 cluster consists of 7 genes, specifically: Dkk2, Vangl2, Lgr6, Fzd6, Fzd4, Fzd8 and Fzd9. The Mcam cluster is comprised of 12 genes: Lrp12, Lgr5, Spon1, Mdb3, Axin2, Lgr4, Dkk4, Axin1, Mcam and Rspon2.

Finally, a cluster named the ‘Dvl1 cluster’, contains genes that primarily exhibit down-regulation which is strongest at 5 days for the crush injury and 5 to 14 days after nerve transection. The Dvl1 cluster is comprised of the following 12 genes: Fzd3, Ror2, Sfrp5, Lrp10, Ptk7, Dkk3, Celsr2, Dvl1, Daam1, PKC, B-catenin and Sfpr1.

### IHC analysis of the Ryk receptor

**Fig 9:**
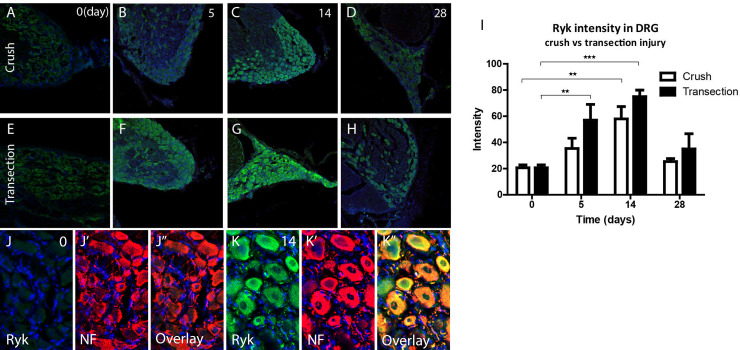
Ryk protein expression is increased in axotomized DRG neurons. A-H, Immunohistochemical staining of Ryk (green) in L5 DRG following sciatic nerve crush (top row) or transection (bottom row) ranging from control (0 day) to 28 days p.i. (right column). Ryk staining intensity is low in control DRG neurons (A, E). Ryk staining intensity increased over time, and peaks at 14d p.i. (C, G) and returns towards baseline at 28d p.i. (D, H). I, Quantitative analysis of Ryk expression in DRG following sciatic nerve lesion (* p<0.05, ** p<0.01, *** p<0.001, error bars represent SEM). J, K, High magnification of DRG tissue sections. Immunohistochemistry for RYK (green) and NF (red) shows that control neurons (J) express very low amounts of RYK (J’). The increase in RYK expression at 14 days following nerve transection (K) is mainly due to an increase in neuronal (NF, K’) expression of RYK (Overlays in J” and K”).

The expression of Wnt receptor, Ryk, was investigated by IHC because of its known involvement in neurite outgrowth during development [15] and neuroregeneration in the spinal cord [16, 17]. Immunohistochemical analysis for Ryk was performed on the DRG following both crush (Fig 9A–9D) and transection/coaptation injury (Fig 9E–9H). Ryk protein expression shows a clear up-regulation, over time in both injury paradigms (Fig 9), which corroborates earlier findings [24]. Double staining for RYK and NF shows that mainly the neurons in the DRG contribute to the increased RYK protein levels observed after nerve injury (Fig 9J non-injured vs. 9K 14 days after transection). A quantitative analysis demonstrates a similar pattern for both injury paradigms. Ryk staining intensity increases both after crush and transection injury and peaks at 14 days for both injury paradigms (crush: p<0.01, transection: p<0.001), while at 28 days p.i. Ryk staining intensity returns to baseline for both injury paradigms (Fig 9I).

## Discussion

In this study the expression of a large proportion of the Wnt pathway genes was investigated in the context of human and rat peripheral nerve injury. In human NIC, Wnt5a is the most prominently up-regulated Wnt-gene. Cells in the NIC express Wnt5a/b and regenerating axons express the Wnt5a/b receptor Ryk. Injury to the sciatic nerve of the rat induced coordinated changes in the expression of the Wnt gene family both in the injured nerve and in the DRG. In the injured rat nerve, four gene clusters could be identified (designated the F-Spon1, Wnt2, Axin2 and MBP clusters) with distinct temporal expression profiles that correspond to particular phases of axon regeneration and post-lesion functional recovery. The DRG exhibits dynamic up- and down regulation of a number of Wnt receptors, which suggests that nerve injury has a significant impact on the responsiveness of injured sensory neurons to Wnt ligands. In the injured rat nerve Wnt5a is up-regulated in the proximal nerve portion and at the injury site, while Wnt5b and Wnt4 are the most prominently up-regulated Wnt ligands at the injury site and in the distal nerve portion. Wnt5a/b protein is expressed in Schwann cells and Ryk is up-regulated in the DRG neurons after nerve injury. Thus, Wnt5a is up-regulated in the human NIC and in the proximal nerve portion and transection injury site of the rat nerve. Wnt5b and Wnt4 are up-regulated at the injury site and in the distal portion of the rat nerve, however these Wnt ligands are expressed but not increased in the NIC. The changes in expression of the Wnt pathway genes both in human NIC and rat PNS injury indicate a central role for Wnt signalling in PNS regeneration.

### Similarities and differences in Wnt5a, Wnt5b and Wnt4 expression in human NIC and in the injured rat nerve

**Fig 10:**
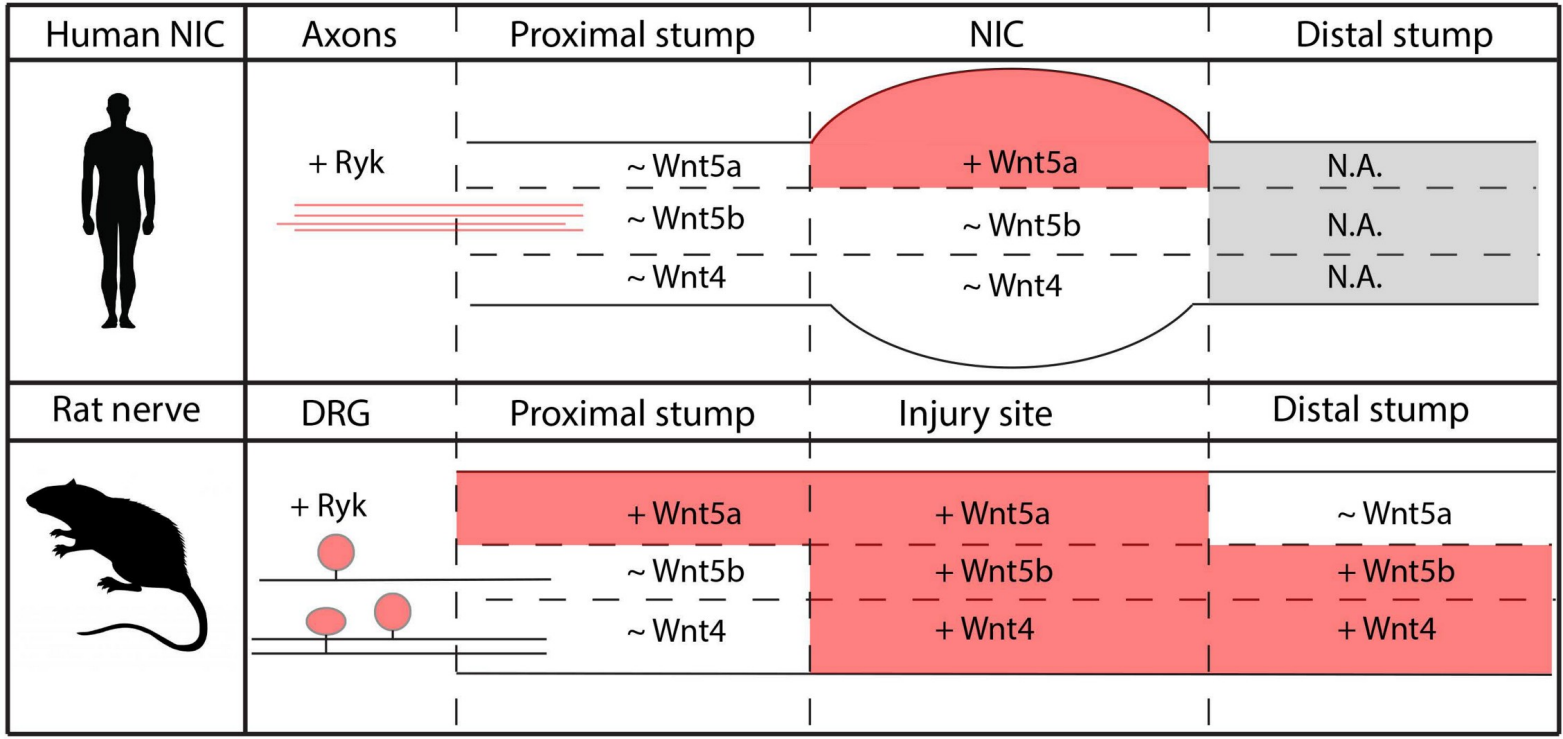
Comparative summary of Wnt5a, 5b and Wnt4 expression in human NIC and the injured rat nerve. Summary of the observations on Wnt5a, Wnt5b and Wnt4 expression in human NIC and in the injured rat sciatic nerve. In NIC tissue Wnt5a mRNA levels are increased (indicated by + and red colour) compared to the proximal stump while Wnt5b and Wnt4 mRNA expression are detectable in the proximal stump and NIC but the levels are not increased (indicate by ~ and no colour). Following injury of the rat nerve Wnt5a mRNA expression is induced in the proximal stump and at the injury site. Wnt5b and Wnt4 mRNA levels are increased at the injury site and the distal stump in comparison to the proximal stump. The Wnt5a receptor RYK is clearly detectable on axons in the NIC and is induced in rat DRG neurons following nerve injury. Human distal nerve stumps were not analysed (N.A.).

The cellular response to injury in a human baby and in an adult rat peripheral nerve is quite different. The formation of a NIC in an injured adult or new born human nerve is the result of an extensive fibrotic response. It has been challenging to induce a NIC in adult rats that resembles the human NIC. Nerve traction combined with a crush injury resulted in subtle fusiform enlargement in rat nerves and a chaotic pattern of axon growth [53, 54]. However, this lesioning procedure did not induce the extensive fibrotic response observed following traction injuries to human nerves. We therefore choose to use the widely used and well-characterized nerve crush and transection as our injury models in the rat. Although the formation of a NIC is not observed in rats after a nerve crush or nerve transection [1, 55], these models do induce Wallerian degeneration and following a transection injury endoneurial tubes are severed, Schwann cells and fibroblasts proliferate and migrate into the lesion site and regenerating axons display a relatively chaotic pattern of growth before they enter an endoneurial tube in the distal nerve stump. Fig 10 summarizes the similarities and differences in Wnt ligand gene expression between the human NIC and injured rat nerve. In the human NIC, Wnt5a is the only significantly up-regulated Wnt ligand, whereas Wnt5b and Wnt4 are expressed in the NIC but expression of these two Wnt ligands is not increased. In the injured rat nerve, Wnt5a is up-regulated in the proximal stump and injury site, whereas Wnt4 and Wnt5b are up-regulated in the injury site and distal nerve stump (Fig 10).

In the human NIC differential expression of several other Wnt genes that act at different levels of the Wnt signalling pathway was observed. In addition to Wnt5a, a Wnt-receptor (Ror1), a Wnt-modulator (Sfrp4) and two downstream Wnt pathway members (Daam1 and Nlk). All these genes are also significantly regulated in the injured rat nerve, but the injured rat nerve exhibits regulation of many more Wnt genes (discussed below).

### A role for Wnt5-Ryk signalling in the injured human and rat nerve?

In both the human NIC and in the injured rat nerve Schwann cells express Wnt5a/b. A diffuse extracellular staining was also observed may be the result of Wnt5a/b into the extracellular space. Regenerating axons in the NIC and injured rat DRG neurons express the Wnt receptor Ryk (Fig 9), which indicates that regenerating axons in the human NIC and in the injured rat nerve could potentially be responsive to Wnt5a/b secreted by Schwann cells. Wnt5a/Ryk signalling has been implicated in axon outgrowth both during development as well as after spinal cord injury. The involvement of Derailed (Drl), a Ryk homologue, in axon guidance was originally discovered in Drosophila [27, 56–59]. During mammalian development non-canonical Wnt signalling mediates axon guidance of several neuronal populations including ventral midbrain monoaminergic neurons [13, 39] and striatal projections [60]. Wnt5a guides axons over the corpus callosum via the Ryk receptor [25] and cortical spinal tract (CST) axons grow through the developing spinal cord driven by an anterior to posterior gradient of Wnt1 and Wnt5a. The development of the CST was impaired by infusion of antibodies that interfered with the function of Ryk [15]. In the spinal cord a re-induction of Wnt gene expression occurred upon injury [16, 17, 26, 61]. Wnt5a is expressed in the glial scar that forms after a spinal cord lesion, while the Wnt receptor Ryk is expressed by injured spinal cord axons and function blocking antibodies to Ryk promoted local sprouting of CST axons [17]. Thus, Wnt/Ryk signalling is implicated in the inhibition of axon regeneration following CNS injury [16, 17, 26]. In the human NIC, Wnt5a/Ryk signalling may therefore also contribute to the aberrant patterns of axon growth and/or the impaired regeneration of axons into the distal nerve stump. Conversely, Ryk-mediated stimulation of neurite outgrowth of embryonal mouse DRG neurons via Wnt3a has also been reported [28]. This implies that, in some situations, Ryk can also be involved in the stimulation of axon outgrowth.

In the distal portion of the injured rat nerve Wnt5b is up-regulated while Wnt5a expression is not increased in the distal nerve portion. The core promoter of Wnt5a is very similar in human and rat (72.5% homology), whereas the Wnt5b promoter region is not conserved between the two species [62]. This may underlie the differential regulation of Wnt5b in the human NIC and at the injury site and in the distal portion of the rat nerve. Wnt5a has a high amino acid homology to Wnt5b (75.8%) [62]. Although Wnt5b signalling has not been extensively studied, Wnt5b also signals through the Ryk receptor in Zebrafish gastrulation [63]. However, Wnt5a and Wnt5b display distinct effects in chondrocyte development [45]. It is therefore difficult to predict if Wnt5a and Wnt5b have similar functions in the injured rat and human nerve. Future studies using bioassays and by *in-vivo* manipulation of Wnt signalling should reveal the specific role of Wnt5a/b and Ryk in axon regeneration.

### Wnt gene expression in the injured rat nerve

Four distinct clusters of Wnt genes were identified in the injured rat nerve. The F-Spon1 cluster consists of three Wnt ligands (Wnt1, Wnt4 and Wnt5b). These genes exhibit early up-regulation and remain up-regulated until 90d p.i. This temporal expression profile suggests that the significantly regulated Wnt ligands in this cluster (Wnt4 and Wnt5b) could potentially be involved in the stimulation of axon regeneration, the activation and de-differentiation of Schwann cells and in regenerative processes that occur later, i.e. remyelination and target cell re-innervation. The potential involvement of the genes in the F-Spon1 cluster in these processes is supported by known functions of Wnt4. Wnt4 (like Wnt5a) has been implied in CST development, Wnt4 attracts sensory neurites through Wnt receptor Fzd3 [15, 64]. Following spinal cord injury Wnt4 is re-expressed around the injury site [17]. Surprisingly, implantation of Wnt4 over-expressing bone marrow stromal cells in the injured spinal cord resulted in long range axon retraction [16]. This demonstrates that during spinal cord development and regeneration Wnt4 has very different effects on neurite growth. Wnt4 also plays a role in regulating the innervation of the neuromuscular junction. Wnt4 null-mutant mice demonstrate profound deficits in muscle innervation [65]. Taken together, this demonstrates that Wnt4 guides sensory neurite growth during CNS development, impedes neurite growth during CNS injury and guides the growth of motor neurites during PNS development. As mentioned before, Wnt5a has also been demonstrated to be involved in these processes [13–17, 21, 22]. The high degree of amino acid homology between Wnt5a and Wnt5b suggest that Wnt5a and Wnt5b may have very similar roles in PNS regeneration. Therefore, the role of Wnt5a, -5b and Wnt4 in PNS regeneration warrants further investigation.

**Fig 11:**
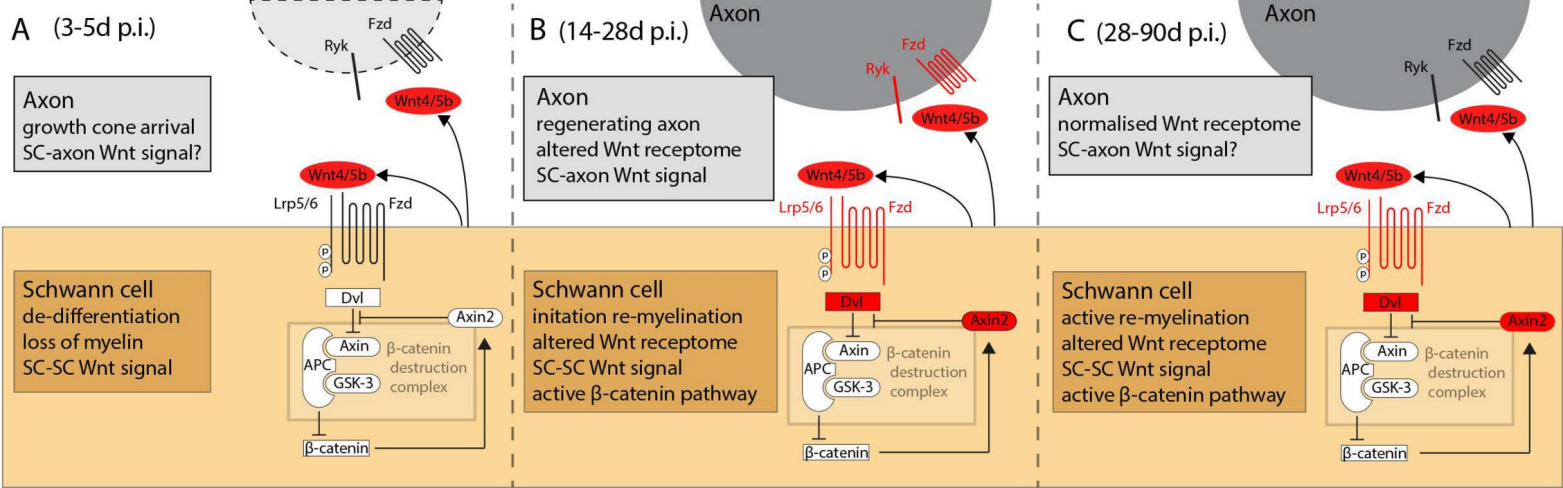
Overview of Wnt gene expression during regeneration in the injured rat nerve. A working model on how Wnt gene expression coincides with regenerative processes in the distal injured rat nerve. Red illustrates up-regulation. *A*, shows (early) denervated Schwann cells (bottom) as found in the injured distal peripheral nerve from 3-5d p.i. Schwann cells de-differentiate and produce Wnt4 and Wnt5b for autocrine and paracrine signalling, and possibly signal to arriving growth cones (top). *B*, shows recently regrown axons (top) at 14–28d p.i. together with Schwann cells (bottom) of the injured nerve. Schwann cells produce Wnt4 and Wnt5b to signal towards regenerating axons that have up-regulated multiple Wnt receptors including Ryk. Wnt5b and Wnt5 also mediate Schwann cell autocrine signalling, at 14d p.i. through altered Wnt receptor availability initiates differentiation and re-myelination due to activation of the β-catenin pathway as demonstrated by Axin2 up- regulation. *C*, exhibits the late regenerative response at 28–90d p.i., with regrown axons (top) and a re-myelinating Schwann cell (bottom). Re- myelinating Schwann cells still produce Wnt4 and Wnt5b but axons have returned their Wnt receptor expression to baseline. Schwann cells signal with Wnt ligands in an autocrine fashion to stimulate the β-catenin pathway as indicated by Axin2 up-regulation.

Schwann cells of the injured nerve dramatically alter their function during the regenerative process. They first de-differentiate and stimulate axonal growth at the early stages of regeneration, and subsequently re-myelinate regenerated axons. The potential involvement of Wnt signalling in the switch from a growth promoting cell to a myelinating cells is indicated by the differential patterns of expression of genes in the MBP and Axin2 clusters which is especially clear in the transected distal nerve (Fig 3C). The MBP cluster consists of genes that are down-regulated at early time points and return to baseline between 14 and 28d p.i. MBP is a principle component of myelin and therefore a marker for mature myelinating Schwann cells. Therefore, genes in the MBP cluster may have roles in the post-lesion maturation of Schwann cells and in Schwann cell myelinisation (Fig 11A). At 14-28d p.i. the Axin2 cluster is up-regulated. This pattern of expression suggests that the genes in this cluster are involved in Schwann cell differentiation and/or re-myelination. Axin2 gene expression is stimulated by the β-catenin Wnt pathway [66, 67], and has therefore been employed as an indicator for active β-catenin Wnt signalling [68] also in Schwann cells [19]. Furthermore, the Axin2 cluster contains numerous genes that are involved in the β-catenin pathway, including Lrp5/6, Fzd1/2/3/4/6/7 and Wnt3a. This all suggests that several genes in the Axin2 cluster are involved in the β-catenin pathway, a pathway essential for lineage progression of neural crest cells [69], the process of axonal sorting [19] and for myelination in Schwann cells [70].

Altered Wnt receptor presence at the plasma membrane determines the outcome of Wnt ligand signalling [14, 29]. The Axin2 cluster includes 15 of the 17 Wnt receptors, indicating that cells of the injured nerve undergo alterations in their responsiveness to Wnt-signalling during later stages of regeneration. Possibly, Schwann cells alter their Wnt receptor expression to shift their interpretation of the Wnt5b and Wnt4 signal from de-differentiation to initialize re-myelination. This changing receptor expression pattern could underlie the activation of different Wnt pathways, in this case from β-catenin independent towards β-catenin dependent pathways on later post-lesion timepoints. This is consistent with earlier findings that show that the β-catenin pathway is an essential driver for myelination in Schwann cells [70] (Fig 11B). In later stages (28-90d p.i.) of the regenerative response Schwann cells continue to re-myelinate and persist their altered Wnt receptor expression, and indeed Axin2 remains up-regulated indicating active β-catenin signalling (Fig 11C).

### Changes in Wnt receptor expression in the DRG

Axotomized DRGs exhibit five Wnt gene clusters. The R-Spondin1 and Ryk clusters contain a number of Wnt receptors which are up-regulated following injury, including Ror1 [71, 72], Lrp5 [73], Fzd1 [74], Fzd2 [75], Fzd5 [76] and Ryk [28]. All of these receptors have been implicated in the process of neurite outgrowth. Of these six receptors it is known that Ryk is a known receptor for Wnt5a, -5b and 4 [15, 16, 63], Ror1 is a Wnt5a receptor [77] and Fzd5 is a receptor for Wnt5a and -4 [78]. This suggests that injured sensory neurons undergo significant changes in their responsiveness to Wnt ligands that are expressed and increased in the injured nerve. The expression of these Wnt receptors peaks at 14d p.i. Therefore, DRG neurons are possibly more responsive to Wnt stimulation at 14d p.i. compared to uninjured DRG neurons (Fig 11B).

In addition to potential alterations in ligand receptor interaction at the level of the regeneration axons, injured DRG neurons may secrete Wnt modulators that affect Schwann cells in the injured nerve. R-spon1 is a ligand for the Lgr4/5 receptor and Lgr4 is up-regulated in the distal stump. Signalling through this receptor results in an increased Wnt receptor presence at the plasma membrane, therefore R-spon1 can function as a Wnt signalling agonist [79–81]. Regenerating sensory neurons could transport R-spon1 and secrete from their regenerating axons to facilitate Wnt signalling in de-differentiated Schwann cells upon contact with a regenerated axon.

## Conclusion and future directions

These findings demonstrate that genes of the Wnt signalling pathway are significantly regulated upon both human and rodent peripheral nerve injury. The regulation of the Wnt pathway suggests that it plays a key role in PNS regeneration. Therefore, further research into the role of Wnt-signalling in PNS regeneration is warranted. As a first step to uncover the various roles of Wnt signalling in PNS regeneration functional *in-vitro* bioassays and *in-vivo* studies in transgenic animals or using viral vector-based strategies could be employed.

## Supporting information

S1 Table Primer list. (DOCX)

S2 Table Fold change and SEM of all genes shown in Fig 3A. (DOCX)

S3 Table Injury. (DOCX)

S4 Table Distal. (DOCX)

S5 Table L5 DRG. (DOCX)
