# Supplementary material for: Coordinated changes in the expression of Wnt pathway genes following human and rat peripheral nerve injury
Source: PLoS One. 2021 Apr 13;16(4):e0249748. doi: 10.1371/journal.pone.0249748 (PMC8043392; doi:10.1371/journal.pone.0249748)
Supplement: S1 Table — (DOCX) [file pone.0249748.s001.docx]

**S1 Table, primer list**

| Primer Name | Primer_Forward (Sense) | Primer_Reverse (AntiSense) |
| --- | --- | --- |
| Fzd1 | CACCTGGATAGGCATCTGGT | ACAGGAAAATAATGGGCCGC |
| Fzd2 | AAGCACGACGGCACCAAGAC | AAGTAGCAGGCGATGACGATGG |
| Fzd3 | CCAGCAGACAGCAGCTTTAG | TGCACAAAGAAATGGCCGAA |
| Fzd4 | AAGGACCAGGTGACGAAGAG | GAACACAGTTCAGGCTCCTTT |
| Fzd5 | CCAGTGTGCCAGGAAATCAC | TGCTTCGTCCTGAGTGTCAT |
| Fzd6 | CAGCTCTCCAGAACCAAACA | CTGAGCGAACAAGCAGAGAT |
| Fzd7 | CATCGGTACGTCCTTCCTGT | CCATGAGCTTCTCCAGCTTC |
| Fzd8 | CTTGTGGTCGGTGCTCTGCTT | AGGTAGAGACAGTGGCGAAGGT |
| Fzd9 | AAGACAGACCCCTCTCTGGA | CCATAACTCAGCCCAGCCTA |
| Fzd10 | AGCACCAGAGGGCTTATTGA | TCTTCCCAAGTGCTCCAACA |
| Lgr4 | ACGCTTAGATGCCAACCATATT | ACAGATGGCGTAACTGGACA |
| Lgr5 | TGAAGCACACTGCTCTGTTG | CGGGACTGATAAACGTGAGG |
| Lgr6 | TACTCTGTAACGGGCTGGTG | ACAAGCTTGACCGGAGACAA |
| Lgr7 | AGTACTGTGGCTATGCACCA | ACACGACCCAGACAAAGACT |
| Lrp5 | CTGGAGCTGCTGCACAGAACAT | CGATGCGGTTGGTCTCTGAGTC |
| Lrp6 | CGTGTTTCACTGGGGACATT | TGAGCTCATCACTGTGGTCT |
| Lrp10 | TGGAACTCACACAGCAGTCC | TCAGGCAAGCAGTGGTTCAT |
| Lrp12 | ATTGCCCAAACGGAAGGGAT | AGTTGCAGCGATCAGAACGA |
| Ror1 | ATCCCACCACAGGGTCAAAT | CTGCGTATCCGGGAGGTATT |
| Ror2 | CAGGACGTGGTGGAGATGAT | TCGATCATGAGGGCGTAGAC |
| Ryk | CACGCAGCTCCAACTACTTC | ACTATGAAGGTGCAAAACTGCT |
| Vangl1 | TTCGTCCTTAAATGCTTGGA | CGGACTGTAAGCGAAGAACA |
| Vangl2 | CACACGCATCGCCAAGGACAT | ACAGCAGTGGAGGCAGCAACA |
| DKK1 | TTCCAACGCGATCAAGAACC | CTTGTTCCCGCCCTCATAGA |
| DKK2 | TGCAAAGTGTGGAAGGATGC | TTCTGGCACACATGGAGTCT |
| DKK2 | TGCTGATGGTGGAGAGTTCAC | CACCTAGAGAGGACTTGATGGA |
| DKK3 | CTCTGAGGTGACCCTGTCAA | TGCACATGGGCTGTGTTATT |
| DKK4 | AGGTGACTGGTAACCGACAA | TATTCTTTGGCATACTCTTAGCCTT |
| Lin28a | TTAAGAAGTCTGCCAAGGGTCT | CTCACTCCCAATACAGAACACG |
| Mcam | AGGAGAGGCAGATACCCATTT | CTGAGGCGGTGTTCATATTCC |
| Nlk | ATGAAGGGCGGCTGAGATAC | TGGTGACAGGCTCAAAGTCA |
| PTK7 | AGAGCACCCACACAGTCAG | AGAGGCCGCTATGTTCGG |
| Rnf43 | TGGAGTCGGAAAGATCAGCAG | ACCTTCCAAGGTGAGGTTCA |
| Wnt1 | ACAGCGTTTATCTTCGCAATCA | TAGTCGCAGGTGCAGGATT |
| Wnt2 | AATTCGCCCGTGCATTTGTA | CAGAGTACAGGAACCGCTCA |
| Wnt2b | CACCCAGTGTGAGTGCAAAT | AGTGTTTCTGCACTCCTTGC |
| Wnt3 | CGCAATTACATCGAGATCA | CATCTATGGTGGTACAGTTC |
| Wnt3a | TCTGCCATGAACCGTCACAACA | AGCAGGTCTTCACTTCGCAACT |
| Wnt4 | AAGGCCATCCTGACACACAT | CGCCGTCAAACTTCTCCTTT |
| Wnt5a | CCACGCAGGACCTGGTCTACAT | TAGCCACGCCCACAGCACAT |
| Wnt5b | TCGACAGAGGCGGTGGAACT | AGGCTCGGCTGATGGCATTC |
| Wnt6 | TCCTCTACGCAGCCGATTCAC | CAACAGGTCGCAGCCGCTAA |
| Wnt7a | ACACTGCCACAATTCCGAGA | ATGGACGGCCTCGTTGTATT |
| Wnt7b | AACACGCACCAGTACACCAAGG | TGACGAAGCAACACCAGTGGAA |
| Wnt9a | CTGTGGGGACAACCTCAAGT | CCCAGGAACTCCTTGACAAAC |
| Wnt9b | CAGCTCTCCCCATTTCGTGA | TTTGACAGCCGTGTCATAGC |
| Wnt10a | CGCACGCATGAGACTCCACAA | TTCGCCGCATGTTCTCCATCAC |
| Wnt10b | ACAAGACCAGCCGCCTATT | CGTTGACCCACTCTGTGACT |
| Wnt11 | TTTCCGATGCTCCTATGAAGGT | CCCCACTTCACTGTTGTGTAGA |
| Wnt16 | AGAGAGTGCAACCGTACGTC | TGTGGACATCGGTCATGCT |
| FoxO1 | TACTCGGCCCAAGGAAAGTT | TTAGCAGAGCACAGGCAGTA |
| FoxO3 | AGAACAGACCAGCCACCTTT | CATCATTGGGTCGTTGCGAA |
| FoxO4 | TAACAGGTCCTCGGAAGGGA | ATATCTGGGCGAGTGTCAGC |
| FoxO6 | CATGCCAGCGACGACTAC | CTTGGGTACATGAGTGGCGA |
| Axin1 | CAAACCAAGCCAGCCACTAA | TCTGTCTGTGCCTGGTCAAA |
| Axin2 | AAACATGACCACTCTGGGCTA | TCCTGTTCGGGCAAGCTAT |
| Celsr2 | ACAGCTGTCGTCTTCCTGTC | ACGTAGCGCTTCTCGCTAAA |
| Ctnnb1 | CCTCTGTGAACTTGCTCAGGA | TTCATTCCTGGAGTGGAGCAA |
| Daam1 | ACTTGGATGGCCTGTCATGTA | AGCCAATGAGGGAGGTATGT |
| Dvl1 | TGGTGGTGCAGCTCAAGTAT | CTGTGCAGTTCTGGGAGACT |
| Dvl2 | ACCCATCTTGAGGCCACATT | TGAGCCCAGGCACTAAGAAG |
| Mbd3 | ACACCTGGCTATGCCTACCT | TATGGGCAGGAGCAGTTCAG |
| Rspo1 | TTGGACCGATGGAGAACCTG | AGGCCAGATCCACCATAACC |
| Rspo2 | TCCCATTTGCAAGGGTTGTTT | ATACTGGCGCATCCCTTCTC |
| Rspo3 | TACTATGCACTTGCGACTGATTT | TTGGCTGCCGATGTATTCCATA |
| Rspo4 | ACATGCGAGAGCTGTTTCAG | AGACACTTCCCTTTGTAGAGGT |
| Spon1 | AAGTCTCCAGGGTCATGGGT | ACGGAAATCACGGCTCACAA |
| Spon2 | ACCGCTGGACTGTGAAGTTT | TAGCGAGTTCTGCTCTTGGC |
| Sfrp1 | CTGCTCAACAAGAACTGCCA | TAGATAGGCCGGTCCAGACA |
| Sfrp2 | CAGAAGGGCCAGAGAGAGTT | TGGGAAACCGGAAATGAGGT |
| Sfrp4 | AAGGTCACCTGCTTCCAAAC | TCCTGTAAGGGTGGCTTCAA |
| Sfrp5 | TAGACAACCTGACAGGGAGC | TTCTTCTTGTCCCAGCGGTA |
| Prkzc | CATCGGGCGTGGAAGCTAT | TCACCACCTTCATGGCGTA |
| Wif1 | TCACACTTGTGTTAGTGGCATTTA | TCCAAGGCAAAGACTCCATACA |
| Znrf3 | TACACACTCACCGAGGAACC | AGCCCAAGTCACAGTCTACA |
