# Supplementary material for: Coordinated changes in the expression of Wnt pathway genes following human and rat peripheral nerve injury
Source: PLoS One. 2021 Apr 13;16(4):e0249748. doi: 10.1371/journal.pone.0249748 (PMC8043392; doi:10.1371/journal.pone.0249748)
Supplement: S4 Table — (DOCX) [file pone.0249748.s004.docx]

**S4 Table. Distal**

| Gene | control | | 3d Crush | | 5d Crush | | 14d Crush | | 28d Crush | | 42d Crush | | 90d Crush | | 3d Trans | | 5d Trans | | 14d Trans | | 28d Trans | | 42d Trans | | 90d Trans | |
| --- | --- | --- | --- | --- | --- | --- | --- | --- | --- | --- | --- | --- | --- | --- | --- | --- | --- | --- | --- | --- | --- | --- | --- | --- | --- | --- |
|  | **mean** | **SEM** | **mean** | **SEM** | **mean** | **SEM** | **mean** | **SEM** | **mean** | **SEM** | **mean** | **SEM** | **mean** | **SEM** | **mean** | **SEM** | **mean** | **SEM** | **mean** | **SEM** | **mean** | **SEM** | **mean** | **SEM** | **mean** | **SEM** |
| Wnt1 | 0 | 0,3 | 14,9 | 0,6 | 3,8 | 0,6 | 11,0 | 0,1 | 7,1 | 1,6 | 6,0 |  | 5,4 | 1,8 | 1,3 | 2,2 | 12,0 |  | 6,9 | 0,1 | 2,2 | 1,4 | 2,4 | 2,8 | 13,0 | 1.0 |
| Wnt2 | 0 | 1,3 | 2,9 | 0,8 | 3,0 | 1,0 | 2,3 | 1,0 | 1,1 | 1,1 | 1,1 | 1,0 | 1,5 | 0,5 | 4,2 | 1,2 | 3,6 | 0,7 | 3,3 | 0,2 | 2,6 | 0,8 | 1,5 | 0,6 | 2,9 | 0.1 |
| Wnt2b | 0 | 1,6 | 0,8 | 1,9 | 1,6 | 4,7 | 2,0 | 2,4 | 1,4 | 1,3 | 1,5 | 3,0 | 1,7 | 1,4 | 0,7 | 2,3 | 1,0 | 1,5 | 1,1 | 4,1 | 2,0 | 1,8 | 1,4 | 2,2 | 1,5 | 0.1 |
| Wnt3 | 0 | 1,6 | 0,1 | 1,9 | 1,3 | 2,6 | 1,3 | 1,2 | 0,2 | 3,9 | 0,1 | 3,5 | 0,3 | 2,0 | 0,2 | 3,9 | 0,2 | 2,3 | 0,7 | 1,4 | 0,7 | 1,5 | 0,1 | 2,5 | 0,2 | 0.7 |
| Wnt3a | 0 | 0,7 | 0,4 | 0,5 | 0,6 | 0,5 | 0,9 | 0,8 | 0,4 | 0,9 | 0,6 | 0,6 | 15,5 | 0,4 | 1,9 | 0,2 | 1,9 | 0,6 | 4,9 | 0,6 | 9,2 | 0,2 | 3,3 | 0,2 | 2,3 | 0.6 |
| Wnt4 | 0 | 0,2 | 3,6 | 0,5 | 4,4 | 0,9 | 15,4 | 0,8 | 3,4 | 1,7 | 2,5 | 0,9 | 4,5 | 0,9 | 3,8 | 0,8 | 3,9 | 1,3 | 12,9 | 0,4 | 16,3 | 0,7 | 6,6 | 0,5 | 6,4 | 0.5 |
| Wnt5a | 0 | 1,9 | 0,4 | 2,7 | 0,8 | 4,1 | 2,6 | 0,6 | 0,8 | 2,7 | 1,1 | 2,9 | 1,8 | 1,2 | 0,5 | 2,8 | 0,5 | 3,2 | 0,8 | 2,1 | 1,5 | 1,9 | 1,0 | 1,4 | 2,9 | 0.2 |
| Wnt5b | 0 | 0,8 | 3,8 | 0,7 | 2,8 | 1,0 | 6,8 | 0,1 | 5,0 | 0,4 | 8,0 | 0,7 | 12,7 | 0,4 | 4,0 | 0,4 | 2,7 | 0,4 | 4,6 | 1,1 | 5,3 | 0,6 | 6,0 | 0,3 | 9,8 | 0.4 |
| Wnt6 | 0 | 3,4 | 0,5 | 2,4 | 0,9 | 9,6 | 1,8 | 5,8 | 1,5 | 7,2 | 1,9 | 5,1 | 1,9 | 4,5 | 0,8 | 2,4 | 0,9 | 7,3 | 1,7 | 10,5 | 3,4 | 4,1 | 4,4 | 1,4 | 2,3 | 0.1 |
| MBP | 0 | 0,5 | 0,1 | 0,6 | 0,1 | 1,1 | 0,9 | 0,6 | 0,9 | 0,8 | 1,2 | 0,8 | 1,0 | 0,3 | 0,1 | 0,4 | 0,0 | 0,6 | 0,1 | 1,1 | 1,3 | 0,4 | 1,7 | 0,2 | 1,6 | 0.1 |
| Wnt7b | 0 | 0,4 | 0,3 | 0,3 | 0,9 | 1,3 | 0,6 | 1,7 | 1,8 | 2,7 | 0,5 | 2,5 | 1,5 | 0,5 | 0,2 | 1,2 | 0,6 | 0,6 | 1,0 | 1,6 | 1,0 | 0,4 | 0,5 | 2,2 | 1,9 | 0.2 |
| Wnt9a | 0 | 3,2 | 2,6 | 6,8 | 2,7 | 9,8 | 2,8 | 3,1 | 1,4 | 5,3 | 1,5 | 8,0 | 1,4 | 5,3 | 1,5 | 7,8 | 1,3 | 5,1 | 1,6 | 8,3 | 1,0 | 6,0 | 1,1 | 1,8 | 2,6 | 0.3 |
| Wnt9b | 0 | 1,1 | 1,1 | 1,2 | 3,5 | 1,6 | 1,4 | 1,2 | 1,9 | 0,9 | 2,4 | 0,5 | 2,4 | 0,7 | 0,9 | 0,8 | 1,0 | 1,0 | 0,4 | 2,2 | 1,4 | 0,7 | 1,1 | 1,7 | 2,0 | 0.2 |
| Wnt10a | 0 | 1,2 | 1,0 | 2,9 | 2,6 | 3,2 | 5,1 | 1,9 | 2,9 | 1,4 | 5,5 | 1,6 | 4,9 | 2,1 | 1,0 | 2,7 | 1,7 | 1,1 | 2,5 | 1,9 | 4,7 | 1,7 | 8,7 | 2,1 | 5,0 | 1.3 |
| Wnt10b | 0 | 0,9 | 1,2 | 0,4 | 2,6 | 0,5 | 4,2 | 0,4 | 2,7 | 0,7 | 2,2 | 0,5 | 3,6 | 0,3 | 1,5 | 0,7 | 2,3 | 0,4 | 2,8 | 0,7 | 5,5 | 0,3 | 2,2 | 0,2 | 5,0 | 0.2 |
| Wnt11 | 0 | 1,0 | 2,0 | 0,5 | 5,4 | 1,8 | 3,6 | 1,0 | 2,0 | 1,7 | 2,3 | 1,8 | 3,2 | 1,1 | 1,7 | 1,4 | 3,2 | 1,5 | 3,0 | 2,9 | 3,7 | 1,3 | 2,3 | 1,7 | 5,1 | 0.2 |
| Fzd1 | 0 | 2,5 | 1,3 | 6,4 | 0,8 | 7,9 | 2,3 | 2,7 | 1,4 | 10,1 | 2,8 | 4,0 | 2,5 | 4,1 | 0,9 | 9,5 | 0,8 | 4,9 | 0,9 | 11,0 | 3,1 | 1,1 | 1,6 | 4,2 | 2,3 | 0.2 |
| Fzd2 | 0 | 1,3 | 1,9 | 2,4 | 1,4 | 4,5 | 2,3 | 1,8 | 1,9 | 1,0 | 2,2 | 3,6 | 2,0 | 2,2 | 1,2 | 0,9 | 2,0 | 2,2 | 3,0 | 3,0 | 4,5 | 2,4 | 2,8 | 1,4 | 2,1 | 0.2 |
| Fzd3 | 0 | 2,1 | 1,5 | 0,9 | 1,3 | 3,8 | 3,5 | 0,9 | 2,7 | 2,0 | 2,4 | 1,3 | 1,7 | 1,2 | 0,9 | 0,5 | 1,3 | 3,0 | 2,0 | 4,1 | 6,2 | 0,6 | 2,7 | 0,7 | 1,9 | 0.1 |
| Fzd4 | 0 | 2,5 | 1,5 | 1,7 | 0,9 | 3,7 | 1,7 | 1,5 | 1,3 | 2,5 | 1,5 | 3,3 | 2,5 | 1,3 | 1,0 | 0,1 | 1,7 | 1,7 | 1,3 | 2,9 | 4,2 | 1,9 | 1,9 | 1,2 | 2,6 | 0.2 |
| Fzd6 | 0 | 1,0 | 1,3 | 1,2 | 2,3 | 4,5 | 2,9 | 0,8 | 1,1 | 3,4 | 1,8 | 3,4 | 1,4 | 2,2 | 1,0 | 1,4 | 1,8 | 2,4 | 1,8 | 3,7 | 2,8 | 0,5 | 1,7 | 0,4 | 1,3 | 0.2 |
| Fzd7 | 0 | 4,3 | 0,8 | 7,0 | 0,8 | 8,0 | 1,5 | 2,2 | 1,2 | 1,1 | 1,7 | 7,2 | 1,7 | 5,0 | 0,5 | 2,9 | 0,8 | 4,7 | 0,7 | 10,6 | 1,9 | 2,7 | 1,4 | 4,9 | 1,9 | 0.2 |
| Fzd8 | 0 | 5,0 | 1,3 | 4,7 | 2,2 | 14,6 | 5,0 | 3,3 | 2,2 | 6,3 | 3,5 | 8,8 | 2,0 | 3,9 | 0,9 | 0,6 | 1,4 | 7,0 | 2,1 | 9,4 | 4,4 | 1,7 | 2,8 | 2,8 | 2,0 | 0.2 |
| Fzd9 | 0 | 1,4 | 0,2 | 0,8 | 0,2 | 1,4 | 0,3 | 1,1 | 0,1 | 1,2 | 0,8 | 0,9 | 1,2 | 0,9 | 0,1 | 2,7 | 0,2 | 1,3 | 0,3 | 0,9 | 0,6 | 0,8 | 1,0 | 2,5 | 1,3 | 0.4 |
| Ror‐1 | 0 | 2,2 | 2,0 | 2,6 | 0,8 | 2,4 | 2,1 | 1,4 | 2,1 | 3,0 | 2,9 | 2,7 | 3,2 | 1,2 | 0,7 | 0,7 | 1,4 | 1,7 | 1,7 | 3,3 | 4,4 | 0,8 | 3,0 | 0,6 | 3,4 | 0.1 |
| Ror-2 | 0 | 2,3 | 2,2 | 0,9 | 1,1 | 2,1 | 1,7 | 1,2 | 1,6 | 2,0 | 1,6 | 1,2 | 1,3 | 1,2 | 1,6 | 0,4 | 1,5 | 1,8 | 2,1 | 1,7 | 2,7 | 1,1 | 2,2 | 1,0 | 1,5 | 0.2 |
| Vangl1 | 0 | 4,4 | 2,1 | 4,3 | 1,9 | 9,2 | 1,4 | 5,5 | 2,4 | 17,7 | 1,5 | 6,8 | 1,5 | 5,9 | 1,5 | 3,1 | 3,7 | 5,0 | 3,1 | 8,8 | 4,7 | 3,3 | 2,2 | 3,7 | 1,6 | 0.3 |
| Vangl2 | 0 | 0,8 | 2,3 | 0,5 | 0,7 | 1,5 | 7,3 | 0,3 | 4,2 | 0,3 | 3,5 | 0,5 | 3,8 | 0,2 | 1,3 | 0,3 | 2,9 | 0,4 | 2,5 | 1,0 | 5,7 | 0,6 | 3,1 | 0,3 | 2,7 | 0.1 |
| Ryk | 0 | 1,8 | 2,7 | 0,6 | 0,9 | 1,5 | 1,9 | 0,6 | 2,4 | 0,4 | 2,6 | 1,0 | 2,3 | 0,5 | 1,7 | 0,2 | 1,9 | 0,7 | 1,5 | 1,5 | 4,8 | 0,4 | 2,6 | 0,5 | 3,4 | 0.3 |
| Lrp5 | 0 | 2,3 | 1,6 | 2,3 | 0,8 | 8,8 | 3,2 | 2,2 | 1,4 | 6,1 | 2,7 | 4,5 | 2,5 | 3,2 | 0,7 | 3,2 | 1,3 | 3,2 | 1,2 | 4,7 | 3,3 | 1,0 | 1,7 | 0,9 | 2,9 | 0.2 |
| Lrp6 | 0 | 2,1 | 1,3 | 0,9 | 1,0 | 1,7 | 1,8 | 0,6 | 1,4 | 1,4 | 2,6 | 1,6 | 2,1 | 0,6 | 0,8 | 0,8 | 1,0 | 0,9 | 1,0 | 1,6 | 2,7 | 0,3 | 1,9 | 0,6 | 2,4 | 0.2 |
| Axin1 | 0 | 3,8 | 1,4 | 5,5 | 3,5 | 6,0 | 5,6 | 2,5 | 1,1 | 1,2 | 1,9 | 5,8 | 1,6 | 4,4 | 1,3 | 5,6 | 2,1 | 3,8 | 1,3 | 6,8 | 3,6 | 2,5 | 2,6 | 3,0 | 4,0 | 0.2 |
| Axin2 | 0 | 1,6 | 0,7 | 0,8 | 2,3 | 1,4 | 4,1 | 0,2 | 1,2 | 0,7 | 1,9 | 0,5 | 1,4 | 1,2 | 0,6 | 0,4 | 2,2 | 0,9 | 1,9 | 1,4 | 5,8 | 0,6 | 3,1 | 0,5 | 4,4 | 0.1 |
| Dvl1 | 0 | 3,4 | 0,5 | 5,2 | 1,3 | 4,8 | 1,8 | 4,1 | 0,5 | 4,0 | 1,3 | 2,3 | 1,2 | 4,5 | 0,7 | 2,5 | 1,5 | 2,3 | 1,3 | 3,9 | 3,6 | 2,1 | 2,0 | 5,5 | 2,5 | 0.1 |
| Dvl2 | 0 | 1,9 | 0,5 | 1,3 | 1,0 | 1,6 | 1,6 | 0,8 | 0,6 | 1,9 | 0,7 | 1,0 | 0,6 | 1,2 | 0,5 | 0,3 | 1,0 | 0,9 | 0,8 | 0,4 | 2,1 | 0,5 | 1,5 | 0,5 | 1,3 | 0.1 |
| Spon1 | 0 | 4,1 | 8,2 | 3,0 | 14,9 | 5,6 | 20,7 | 2,4 | 3,1 | 6,6 | 3,6 | 3,7 | 4,1 | 3,8 | 13,0 | 0,4 | 29,1 | 4,4 | 19,8 | 3,8 | 34,0 | 5,9 | 11,1 | 3,3 | 9,2 | 0.2 |
| Spon2 | 0 | 2,4 | 0,3 | 3,2 | 0,9 | 3,2 | 0,9 | 2,4 | 0,5 | 1,2 | 0,8 | 0,8 | 0,8 | 2,2 | 0,4 | 1,5 | 0,9 | 3,3 | 0,5 | 1,6 | 1,6 | 0,5 | 1,8 | 2,1 | 1,9 | 0.1 |
| Rspon1 | 0 | 1,6 | 0,2 | 0,6 | 0,6 | 1,0 | 1,3 | 0,5 | 0,5 | 1,6 | 0,6 | 0,7 | 0,3 | 1,2 | 0,1 | 0,5 | 0,2 | 0,8 | 0,4 | 1,3 | 0,9 | 0,5 | 0,8 | 0,6 | 1,0 | 0.1 |
| Rspon2 | 0 | 9,2 | 0,2 | 31,8 | 0,4 | 26,6 | 1,0 | 7,3 | 0,1 | 27,2 | 0,3 | 24,5 | 0,2 | 25,7 | 0,5 | 8,3 | 0,9 | 6,5 | 0,5 | 10,1 | 1,3 | 5,6 | 1,0 | 11,0 | 0,6 | 1.4 |
| Rspon3 | 0 | 1,9 | 0,3 | 1,4 | 0,7 | 1,8 | 1,1 | 1,3 | 0,5 | 2,1 | 0,5 | 1,9 | 0,4 | 2,8 | 0,2 | 2,3 | 0,6 | 1,2 | 0,6 | 2,2 | 1,8 | 1,0 | 1,0 | 1,2 | 1,0 | 0.2 |
| Ctnnb1 | 0 | 6,4 | 0,8 | 6,8 | 2,4 | 8,2 | 3,3 | 4,2 | 0,5 | 9,7 | 1,1 | 6,7 | 1,0 | 8,9 | 0,9 | 2,9 | 2,2 | 4,4 | 1,2 | 7,3 | 3,4 | 3,5 | 1,8 | 3,2 | 2,1 | 0.1 |
| Mbd3 | 0 | 2,7 | 1,0 | 2,3 | 1,4 | 3,1 | 1,3 | 1,6 | 0,9 | 6,2 | 0,7 | 0,7 | 0,8 | 2,5 | 1,1 | 1,6 | 1,5 | 0,8 | 1,2 | 0,9 | 3,8 | 0,5 | 2,0 | 0,9 | 1,2 | 0.3 |
| Lrp10 | 0 | 3,8 | 1,2 | 3,4 | 1,9 | 5,1 | 2,0 | 2,7 | 1,0 | 8,4 | 1,0 | 1,3 | 1,2 | 4,7 | 1,5 | 1,5 | 2,4 | 1,0 | 1,5 | 3,0 | 4,4 | 1,0 | 2,8 | 0,7 | 1,7 | 0.2 |
| Lrp12 | 0 | 1,2 | 1,6 | 1,0 | 2,9 | 1,7 | 2,8 | 1,0 | 0,9 | 0,6 | 1,1 | 1,3 | 1,0 | 1,2 | 1,2 | 0,4 | 2,4 | 1,1 | 1,2 | 1,7 | 3,7 | 0,5 | 1,4 | 0,9 | 2,3 | 0.2 |
| Lgr4 | 0 | 2,4 | 0,6 | 9,2 | 1,5 | 9,3 | 2,5 | 3,9 | 0,6 | 3,7 | 1,1 | 6,8 | 0,9 | 8,0 | 0,8 | 2,1 | 1,1 | 6,5 | 0,7 | 5,1 | 2,6 | 1,9 | 1,8 | 2,5 | 2,0 | 0.1 |
| Lgr5 | 0 | 1,9 | 0,1 | 1,7 | 0,2 | 3,9 | 0,4 | 2,2 | 0,1 | 4,1 | 0,4 | 2,4 | 0,4 | 2,6 | 0,1 | 1,3 | 0,1 | 1,3 | 0,1 | 2,2 | 0,4 | 1,6 | 0,3 | 1,7 | 0,5 | 0.2 |
| Lgr6 | 0 | 1,1 | 0,1 | 1,0 | 0,3 | 1,3 | 0,1 | 0,7 | 0,3 | 4,6 | 0,2 | 0,2 | 0,3 | 0,8 | 0,2 | 1,3 | 0,3 | 0,6 | 0,3 | 1,9 | 0,4 | 1,0 | 0,5 | 0,5 | 0,2 | 0.4 |
| Lgr7 | 0 | 1,4 | 0,1 | 0,5 | 0,2 | 1,4 | 0,4 | 0,8 | 0,4 | 1,0 | 0,3 | 0,9 | 0,2 | 0,8 | 0,1 | 0,5 | 0,1 | 0,4 | 0,1 | 0,9 | 1,1 | 1,1 | 0,3 | 0,8 | 0,8 | 0.1 |
| Sfrp1 | 0 | 2,9 | 1,3 | 3,7 | 2,1 | 8,0 | 1,7 | 3,8 | 0,7 | 2,4 | 1,3 | 4,2 | 1,0 | 5,4 | 1,5 | 2,0 | 2,1 | 6,9 | 1,2 | 2,7 | 2,5 | 0,9 | 2,0 | 1,5 | 1,9 | 0.1 |
| Sfrp2 | 0 | 2,2 | 1,5 | 1,3 | 2,1 | 2,2 | 1,6 | 1,6 | 0,5 | 3,6 | 0,7 | 1,9 | 0,8 | 2,5 | 2,4 | 0,9 | 4,2 | 2,0 | 1,5 | 2,1 | 3,1 | 0,7 | 1,2 | 1,6 | 1,5 | 0.2 |
| Sfrp4 | 0 | 1,5 | 2,4 | 0,9 | 2,9 | 1,1 | 0,9 | 1,6 | 0,6 | 1,8 | 0,5 | 1,3 | 0,5 | 0,8 | 3,1 | 0,6 | 9,7 | 1,1 | 2,0 | 1,2 | 3,1 | 0,7 | 1,0 | 0,8 | 1,2 | 0.3 |
| Sfrp5 | 0 | 1,3 | 0,1 | 0,4 | 0,3 | 1,0 | 0,4 | 0,4 | 0,1 | 3,1 | 0,4 | 0,3 | 0,5 | 0,7 | 0,1 | 0,3 | 0,2 | 0,4 | 0,2 | 0,3 | 0,8 | 0,1 | 0,6 | 0,1 | 1,0 | 0.1 |
| Prkzc | 0 | 2,0 | 0,7 | 3,3 | 1,1 | 3,6 | 1,6 | 2,4 | 0,5 | 2,4 | 0,7 | 3,2 | 0,8 | 2,3 | 0,6 | 1,0 | 0,5 | 3,7 | 0,5 | 2,6 | 2,1 | 1,3 | 2,1 | 1,5 | 1,8 | 0.1 |
| Mcam | 0 | 3,0 | 1,0 | 4,0 | 2,0 | 7,2 | 5,6 | 2,4 | 1,3 | 1,5 | 2,0 | 5,6 | 1,2 | 3,9 | 0,9 | 2,6 | 1,2 | 2,6 | 1,5 | 3,0 | 6,2 | 2,0 | 4,8 | 2,9 | 2,9 | 0.1 |
| Celsr2 | 0 | 1,8 | 0,3 | 1,9 | 2,0 | 3,3 | 5,3 | 1,1 | 0,8 | 2,5 | 1,1 | 2,6 | 0,8 | 3,0 | 0,2 | 2,2 | 1,4 | 1,5 | 1,2 | 3,1 | 5,5 | 0,5 | 3,1 | 1,3 | 1,3 | 0.1 |
| Damm1 | 0 | 4,0 | 1,0 | 10,6 | 0,9 | 9,6 | 2,0 | 6,7 | 0,3 | 7,2 | 0,6 | 10,3 | 0,8 | 7,9 | 0,7 | 4,7 | 0,6 | 6,5 | 0,4 | 10,1 | 2,1 | 1,8 | 2,1 | 4,2 | 2,9 | 0.3 |
| PTK7 | 0 | 1,3 | 1,3 | 4,3 | 2,2 | 4,7 | 6,7 | 4,9 | 2,3 | 13,5 | 1,5 | 4,5 | 1,1 | 1,4 | 1,0 | 2,2 | 1,1 | 3,4 | 1,3 | 2,1 | 4,1 | 1,6 | 2,8 | 2,7 | 2,3 | 0.2 |
| DKK2 | 0 | 1,9 | 0,5 | 2,0 | 1,1 | 2,9 | 1,4 | 2,8 | 0,7 | 8,0 | 0,5 | 3,4 | 0,4 | 2,1 | 0,6 | 2,6 | 0,8 | 1,2 | 0,3 | 2,9 | 1,4 | 1,7 | 1,2 | 1,8 | 1,4 | 0.3 |
| DKK3 | 0 | 2,5 | 1,0 | 3,9 | 1,2 | 4,0 | 3,4 | 1,5 | 0,9 | 6,8 | 0,8 | 3,1 | 0,6 | 2,3 | 1,0 | 2,4 | 1,0 | 3,8 | 0,9 | 1,2 | 2,3 | 0,7 | 2,0 | 1,8 | 1,5 | 0.0 |
| DKK4 | 0 | 1,3 | 1,0 | 1,2 | 1,3 | 1,6 | 2,8 | 1,0 | 1,4 | 3,8 | 1,1 | 1,0 | 0,9 | 1,9 | 0,8 | 0,5 | 1,3 | 0,5 | 0,9 | 1,1 | 3,4 | 0,5 | 3,1 | 0,8 | 1,8 | 0.1 |
| Fzd5 | 0 | 1,1 | 0,4 | 0,9 | 0,8 | 1,1 | 1,0 | 1,2 | 1,0 | 3,1 | 0,6 | 1,1 | 0,5 | 0,9 | 0,2 | 2,0 | 0,4 | 1,4 | 0,3 | 1,3 | 0,8 | 1,0 | 0,7 | 0,1 | 0,7 | 0.2 |
| Fzd10 | 0 | 1,2 | 0,9 | 0,8 | 0,7 | 1,7 | 1,5 | 0,4 | 1,4 | 1,3 | 0,4 | 1,1 | 1,1 | 0,5 | 0,4 | 0,9 | 1,8 | 1,0 | 1,0 | 0,4 | 0,8 | 1,6 | 0,8 | 0,8 | 0,4 | 0.5 |
| FoxO1 | 0 | 3,2 | 1,1 | 3,9 | 1,0 | 5,6 | 2,3 | 2,7 | 0,5 | 3,5 | 1,1 | 3,5 | 1,4 | 3,0 | 0,6 | 1,7 | 1,0 | 1,5 | 0,6 | 4,1 | 3,4 | 2,1 | 2,5 | 1,1 | 3,3 | 0.1 |
| FoxO3 | 0 | 3,0 | 1,8 | 4,3 | 1,2 | 7,1 | 4,9 | 2,5 | 0,7 | 1,2 | 1,4 | 5,1 | 1,2 | 2,5 | 0,7 | 2,3 | 0,6 | 3,5 | 0,7 | 5,0 | 3,9 | 1,8 | 2,7 | 2,1 | 3,9 | 0.2 |
| FoxO4 | 0 | 2,1 | 0,5 | 10,4 | 0,6 | 10,4 | 1,4 | 3,9 | 0,3 | 6,7 | 0,8 | 11,4 | 0,6 | 8,4 | 0,2 | 3,7 | 0,3 | 7,1 | 0,3 | 10,8 | 1,0 | 11,4 | 1,1 | 7,2 | 1,4 | 0.2 |
| FoxO6 | 0 | 1,4 | 0,4 | 1,2 | 0,7 | 0,7 | 1,0 | 0,3 | 0,7 | 1,2 | 0,8 | 0,2 | 1,4 | 0,5 | 0,2 | 1,1 | 0,2 | 1,4 | 0,1 | 0,9 | 0,8 | 0,6 | 0,7 | 0,9 | 2,2 | 0.2 |
| Lin28a | 0 | 0,7 | 0,1 | 0,9 | 0,3 | 0,7 | 0,7 | 0,3 | 0,3 | 0,2 | 0,0 | 0,6 | 0,4 | 0,1 | 0,1 | 0,5 | 0,3 | 0,6 | 0,2 | 0,6 | 0,8 | 0,4 | 0,9 | 0,3 | 0,1 | 1.0 |
| Nlk | 0 | 1,2 | 1,0 | 0,6 | 1,0 | 1,6 | 2,3 | 0,8 | 0,7 | 1,0 | 0,8 | 1,1 | 0,7 | 0,8 | 0,5 | 0,3 | 0,8 | 0,4 | 0,8 | 0,8 | 2,9 | 0,5 | 1,6 | 0,3 | 1,4 | 0.0 |
| Rnf43 | 0 | 0,5 | 0,5 | 0,4 | 0,6 | 0,3 | 1,0 | 0,6 | 0,4 | 2,0 | 0,7 | 0,6 | 1,1 | 0,7 | 0,6 | 1,3 | 0,6 | 0,9 | 1,2 | 0,8 | 2,3 | 0,3 | 1,9 | 1,0 | 1,4 | 1.3 |
| Wif1 | 0 | 1,6 | 0,3 | 0,9 | 0,3 | 1,1 | 0,4 | 0,8 | 0,3 | 3,6 | 0,2 | 0,4 | 0,3 | 1,1 | 0,3 | 0,4 | 0,5 | 0,7 | 0,5 | 1,2 | 1,0 | 0,3 | 0,7 | 0,2 | 0,3 | 0.3 |
| Wnt16 | 0 | 2,1 | 0,9 | 1,3 | 0,8 | 3,6 | 2,0 | 1,0 | 0,6 | 1,6 | 0,9 | 2,7 | 0,6 | 1,0 | 0,3 | 1,1 | 0,4 | 1,5 | 1,1 | 3,2 | 3,5 | 1,5 | 2,8 | 0,9 | 1,5 | 0.3 |
| Znrf3 | 0 | 1,4 | 0,7 | 2,3 | 0,5 | 2,5 | 2,6 | 2,1 | 0,7 | 4,4 | 0,8 | 2,5 | 0,9 | 2,5 | 0,3 | 2,6 | 0,2 | 3,5 | 0,3 | 2,0 | 2,4 | 1,2 | 1,7 | 1,6 | 2,7 | 0.1 |
