# Supplementary material for: Coordinated changes in the expression of Wnt pathway genes following human and rat peripheral nerve injury
Source: PLoS One. 2021 Apr 13;16(4):e0249748. doi: 10.1371/journal.pone.0249748 (PMC8043392; doi:10.1371/journal.pone.0249748)
Supplement: S5 Table — (DOCX) [file pone.0249748.s005.docx]

**S5 Table. L5 DRG**

| Gene | control | | 3d Crush | | 5d Crush | | 14d Crush | | 28d Crush | | 42d Crush | | 90d Crush | | 3d Trans | | 5d Trans | | 14d Trans | | 28d Trans | | 42d Trans | | 90d Trans | |
| --- | --- | --- | --- | --- | --- | --- | --- | --- | --- | --- | --- | --- | --- | --- | --- | --- | --- | --- | --- | --- | --- | --- | --- | --- | --- | --- |
|  | **mean** | **SEM** | **mean** | **SEM** | **mean** | **SEM** | **mean** | **SEM** | **mean** | **SEM** | **mean** | **SEM** | **mean** | **SEM** | **mean** | **SEM** | **mean** | **SEM** | **mean** | **SEM** | **mean** | **SEM** | **mean** | **SEM** | **mean** | **SEM** |
| Fzd1 | 0 | 0,1 | 2,2 | 0,3 | 1,8 | 0,4 | 3,4 | 1,4 | 2,1 | 0,1 | 1,6 | 0,1 | 1,8 | 0,2 | 1,2 | 0,2 | 1,5 | 0,2 | 5,6 | 1,1 | 2,1 | 0,1 | 1,6 | 0,2 | 1,5 | 0,1 |
| Fzd2 | 0 | 0,2 | 1,8 | 0,4 | 2,4 | 0,5 | 2,1 | 0,7 | 1,3 | 0,1 | 1,5 | 0,2 | 1,2 | 0,1 | 1,4 | 0,3 | 1,7 | 0,1 | 3,6 | 0,7 | 1,7 | 0,1 | 2,0 | 0,4 | 1,4 | 0,2 |
| Fzd3 | 0 | 0,0 | 1,2 | 0,0 | 0,7 | 0,1 | 0,9 | 0,1 | 1,2 | 0,1 | 0,9 | 0,1 | 1,0 | 0,1 | 0,8 | 0,1 | 0,8 | 0,1 | 1,0 | 0,1 | 1,1 | 0,0 | 0,8 | 0,1 | 1,2 | 0,1 |
| Fzd4 | 0 | 0,2 | 0,8 | 0,1 | 0,7 | 0,2 | 1,0 | 0,3 | 0,9 | 0,1 | 0,6 | 0,1 | 1,2 | 0,2 | 0,6 | 0,1 | 0,8 | 0,2 | 1,8 | 0,9 | 1,0 | 0,1 | 0,6 | 0,1 | 0,8 | 0,2 |
| Fzd5 | 0 | 0,1 | 2,2 | 0,3 | 1,7 | 0,2 | 5,2 | 0,6 | 2,3 | 0,1 | 0,9 | 0,2 | 2,0 | 0,2 | 1,1 | 0,1 | 1,5 | 0,2 | 2,0 | 0,8 | 0,5 | 0,1 | 2,4 | 0,3 | 1,7 | 0,2 |
| Fzd6 | 0 | 0,2 | 0,7 | 0,1 | 1,2 | 0,4 | 1,2 | 0,8 | 0,6 | 0,0 | 0,6 | 0,1 | 0,8 | 0,1 | 0,6 | 0,2 | 0,5 | 0,2 | 3,1 | 2,4 | 0,6 | 0,1 | 0,7 | 0,2 | 0,7 | 0,1 |
| Fzd7 | 0 | 0,1 | 1,2 | 0,2 | 1,4 | 0,2 | 2,2 | 0,7 | 1,6 | 0,1 | 1,6 | 0,2 | 2,7 | 0,3 | 1,0 | 0,2 | 1,7 | 0,1 | 4,5 | 1,0 | 1,7 | 0,1 | 1,7 | 0,2 | 1,5 | 0,1 |
| Fzd8 | 0 | 0,3 | 1,0 | 0,1 | 0,9 | 0,3 | 1,4 | 0,9 | 0,9 | 0,1 | 0,7 | 0,3 | 1,1 | 0,3 | 0,6 | 0,2 | 0,5 | 0,2 | 1,1 | 0,5 | 0,7 | 0,0 | 0,7 | 0,1 | 0,9 | 0,1 |
| Fzd9 | 0 | 0,5 | 0,4 | 0,4 | 0,5 | 0,5 | 0,4 | 0,2 | 0,4 | 0,4 | 0,5 | 0,3 | 0,7 | 0,3 | 0,5 | 0,2 | 0,5 | 0,3 | 0,9 | 0,5 | 0,4 | 0,4 | 0,6 | 0,4 | 0,7 | 0,3 |
| Fzd10 | 0 | 0,3 | 2,8 | 0,2 | 3,1 | 0,3 | 3,4 | 1,2 | 0,0 |  | 1,1 | 0,2 | 2,0 | 0,1 | 0,2 |  | 1,4 | 0,1 | 3,2 | 0,4 | 0,7 | 0,3 | 2,4 | 0,2 | 3,7 | 0,6 |
| Ror-1 | 0 | 0,1 | 1,3 | 0,2 | 3,5 | 0,4 | 5,9 | 1,5 | 2,3 | 0,4 | 2,6 | 0,1 | 2,3 | 0,2 | 1,9 | 0,3 | 2,2 | 0,2 | 5,5 | 0,5 | 2,6 | 0,1 | 1,4 | 0,1 | 2,6 | 0,2 |
| Ror-2 | 0 | 0,5 | 0,8 | 0,2 | 0,8 | 0,0 | 0,8 | 0,1 | 0,7 | 0,1 | 1,3 | 0,2 | 1,3 | 0,3 | 0,8 | 0,1 | 1,4 | 0,4 | 0,8 | 0,2 | 1,0 | 0,1 | 0,7 | 0,1 | 1,3 | 0,2 |
| Vangl1 | 0 | 0,1 | 0,9 | 0,1 | 1,4 | 0,1 | 1,5 | 0,3 | 1,7 | 0,4 | 1,4 | 0,1 | 1,4 | 0,0 | 1,1 | 0,1 | 1,6 | 0,1 | 1,8 | 0,2 | 1,1 | 0,1 | 1,0 | 0,1 | 1,0 | 0,1 |
| Vangl2 | 0 | 0,0 | 0,6 | 0,0 | 1,1 | 0,1 | 0,7 | 0,1 | 2,2 | 0,2 | 0,5 | 0,0 | 0,9 | 0,0 | 0,4 | 0,0 | 1,2 | 0,0 | 1,4 | 0,1 | 0,6 | 0,0 | 0,8 | 0,0 | 0,5 | 0,0 |
| Ryk | 0 | 0,1 | 1,4 | 0,1 | 2,4 | 0,3 | 1,3 | 0,2 | 1,1 | 0,1 | 1,5 | 0,1 | 2,5 | 0,1 | 1,1 | 0,1 | 1,9 | 0,1 | 2,2 | 0,2 | 2,0 | 0,1 | 0,8 | 0,1 | 1,7 | 0,1 |
| Lrp5 | 0 | 0,1 | 3,2 | 0,1 | 2,6 | 0,4 | 2,1 | 0,3 | 3,9 | 0,2 | 1,9 | 0,2 | 4,4 | 0,3 | 2,3 | 0,1 | 1,9 | 0,1 | 7,5 | 0,9 | 2,8 | 0,1 | 2,6 | 0,2 | 2,5 | 0,0 |
| Lrp6 | 0 | 0,2 | 1,5 | 0,2 | 2,0 | 0,6 | 2,3 | 0,9 | 1,4 | 0,1 | 2,5 | 0,1 | 2,3 | 0,1 | 1,2 | 0,2 | 1,2 | 0,1 | 5,3 | 1,8 | 1,8 | 0,2 | 1,2 | 0,1 | 1,9 | 0,1 |
| Axin1 | 0 | 0,1 | 1,2 | 0,2 | 0,5 | 0,2 | 1,4 | 0,3 | 0,8 | 0,1 | 1,0 | 0,3 | 0,7 | 0,1 | 0,7 | 0,2 | 0,5 | 0,1 | 0,9 | 0,2 | 0,9 | 0,0 | 0,6 | 0,2 | 0,8 | 0,1 |
| Axin2 | 0 | 0,2 | 1,4 | 0,4 | 0,5 | 0,1 | 2,0 | 0,6 | 1,8 | 0,1 | 1,2 | 0,2 | 0,7 | 0,1 | 0,7 | 0,2 | 0,8 | 0,1 | 0,6 | 0,1 | 1,2 | 0,1 | 0,8 | 0,1 | 0,7 | 0,0 |
| Dvl1 | 0 | 0,1 | 1,2 | 0,2 | 0,4 | 0,1 | 1,3 | 0,2 | 0,7 | 0,1 | 1,0 | 0,2 | 0,5 | 0,1 | 0,7 | 0,2 | 0,5 | 0,0 | 0,4 | 0,0 | 0,7 | 0,1 | 0,5 | 0,1 | 0,3 | 0,0 |
| Dvl2 | 0 | 0,1 | 1,9 | 0,2 | 1,0 | 0,2 | 5,1 | 1,0 | 2,8 | 0,0 | 1,4 | 0,2 | 1,2 | 0,1 | 1,3 | 0,2 | 1,1 | 0,1 | 1,1 | 0,3 | 0,9 | 0,1 | 1,7 | 0,2 | 1,2 | 0,1 |
| Spon1 | 0 | 0,1 | 1,8 | 0,2 | 0,9 | 0,2 | 2,0 | 0,2 | 1,3 | 0,1 | 1,3 | 0,3 | 0,7 | 0,0 | 1,3 | 0,2 | 1,0 | 0,1 | 0,7 | 0,1 | 1,2 | 0,1 | 1,2 | 0,1 | 0,7 | 0,1 |
| Spon2 | 0 | 0,2 | 1,9 | 0,2 | 0,6 | 0,2 | 2,6 | 0,4 | 0,8 | 0,1 | 1,2 | 0,4 | 1,7 | 0,4 | 1,2 | 0,2 | 1,1 | 0,1 | 1,4 | 0,1 | 1,1 | 0,0 | 1,5 | 0,3 | 1,2 | 0,0 |
| Rspon1 | 0 | 0,0 | 4,8 | 0,4 | 2,2 | 0,1 | 22,1 | 0,7 | 23,2 | 0,1 | 1,4 | 0,0 | 2,7 | 0,0 | 2,1 | 0,1 | 6,3 | 0,6 | 2,6 | 0,3 | 1,3 | 0,1 | 3,9 | 0,1 | 3,4 | 0,1 |
| Rspon2 | 0 | 0,1 | 1,2 | 0,1 | 0,6 | 0,0 | 1,5 | 0,3 | 0,9 | 0,0 | 0,7 | 0,1 | 0,6 | 0,0 | 0,7 | 0,1 | 0,5 | 0,1 | 0,6 | 0,1 | 0,9 | 0,0 | 0,8 | 0,1 | 0,7 | 0,1 |
| Rspon3 | 0 | 0,1 | 1,8 | 0,0 | 2,1 | 0,4 | 4,0 | 0,7 | 2,2 | 0,1 | 1,2 | 0,2 | 1,1 | 0,1 | 0,9 | 0,2 | 1,2 | 0,1 | 2,9 | 1,3 | 1,0 | 0,1 | 1,1 | 0,1 | 1,3 | 0,1 |
| Rspon4 | 0 | 0,0 | 0,7 | 0,1 | 0,8 | 0,3 | 2,6 | 0,5 | 0,5 | 0,0 | 1,5 |  | 3,0 | 0,3 | 1,5 | 0,3 | 1,1 | 0,2 | 0,8 | 0,4 | 1,0 | 0,2 | 2,8 | 1,1 | 0,6 | 0,2 |
| Ctnnb1 | 0 | 0,1 | 1,1 | 0,1 | 0,5 | 0,2 | 1,2 | 0,3 | 0,8 | 0,1 | 0,8 | 0,3 | 0,7 | 0,2 | 0,6 | 0,2 | 0,6 | 0,1 | 0,5 | 0,1 | 0,7 | 0,0 | 0,6 | 0,1 | 0,5 | 0,0 |
| Mbd3 | 0 | 0,1 | 1,3 | 0,3 | 0,8 | 0,2 | 2,4 | 0,7 | 1,1 | 0,1 | 1,3 | 0,4 | 0,7 | 0,1 | 0,8 | 0,1 | 0,9 | 0,1 | 0,6 | 0,1 | 0,7 | 0,0 | 1,0 | 0,1 | 0,7 | 0,1 |
| Lrp10 | 0 | 0,1 | 1,4 | 0,2 | 0,8 | 0,2 | 0,2 | 0,1 | 0,9 | 0,0 | 1,1 | 0,3 | 0,6 | 0,2 | 0,9 | 0,1 | 0,8 | 0,1 | 0,6 | 0,0 | 0,8 | 0,1 | 1,1 | 0,1 | 0,8 | 0,0 |
| Lrp12 | 0 | 0,2 | 2,2 | 0,2 | 0,6 | 0,1 | 2,0 | 0,2 | 1,1 | 0,1 | 1,2 | 0,2 | 0,7 | 0,1 | 1,2 | 0,1 | 0,7 | 0,1 | 1,0 | 0,1 | 1,5 | 0,1 | 1,0 | 0,1 | 1,0 | 0,1 |
| Lgr4 | 0 | 0,1 | 1,4 | 0,1 | 0,5 | 0,0 | 1,0 | 0,3 | 1,1 | 0,1 | 0,9 | 0,3 | 0,9 | 0,2 | 0,8 | 0,2 | 0,6 | 0,1 | 0,6 | 0,1 | 0,9 | 0,1 | 0,6 | 0,2 | 0,8 | 0,1 |
| Lgr5 | 0 | 0,1 | 1,4 | 0,2 | 0,6 | 0,1 | 1,9 | 0,3 | 0,9 | 0,1 | 1,2 | 0,5 | 1,1 | 0,3 | 0,8 | 0,1 | 0,7 | 0,1 | 0,6 | 0,1 | 1,3 | 0,1 | 1,2 | 0,3 | 1,0 | 0,1 |
| Lgr6 | 0 | 0,2 | 0,6 | 0,2 | 0,9 | 0,2 | 2,0 | 0,4 | 3,4 | 0,1 | 0,5 | 0,1 | 1,3 | 0,1 | 0,5 | 0,0 | 0,8 | 0,3 | 1,0 | 0,4 | 0,3 | 0,1 | 0,5 | 0,1 | 0,4 | 0,1 |
| Lgr7 | 0 | 0,0 | 1,7 | 0,1 | 0,5 | 0,0 | 38,5 | 1,7 | 2,0 | 0,0 | 7,8 | 0,2 | 9,4 | 0,0 | 0,5 | 0,0 | 4,3 | 0,2 | 0,6 | 0,0 | 1,0 | 0,0 | 3,6 | 0,1 | 4,2 | 0,1 |
| Sfrp1 | 0 | 0,2 | 1,1 | 0,1 | 0,5 | 0,2 | 1,3 | 0,5 | 0,8 | 0,1 | 0,6 | 0,4 | 0,6 | 0,1 | 0,7 | 0,2 | 0,6 | 0,1 | 0,5 | 0,1 | 0,7 | 0,1 | 0,5 | 0,2 | 0,6 | 0,1 |
| Sfrp2 | 0 | 0,2 | 0,7 | 0,1 | 0,2 | 0,1 | 0,0 | 0,0 | 3,8 | 0,1 | 0,2 | 0,2 | 0,3 | 0,1 | 0,3 | 0,1 | 0,6 | 0,1 | 0,6 | 0,3 | 0,8 | 0,1 | 0,3 | 0,1 | 0,5 | 0,2 |
| Sfrp4 | 0 | 0,1 | 4,9 | 0,2 | 0,9 | 0,1 | 9,1 | 1,1 | 17,2 | 0,0 | 5,8 | 0,2 | 1,4 | 0,1 | 1,7 | 0,1 | 2,7 | 0,1 | 5,8 | 0,6 | 5,1 | 0,2 | 2,0 | 0,1 | 1,4 | 0,1 |
| Sfrp5 | 0 | 0,1 | 1,8 | 0,1 | 0,7 | 0,1 | 0,4 | 0,1 | 0,9 | 0,1 | 0,7 | 0,6 | 1,3 | 0,3 | 0,9 | 0,2 | 0,9 | 0,1 | 0,8 | 0,1 | 1,0 | 0,1 | 1,1 | 0,2 | 0,8 | 0,0 |
| Prkzc | 0 | 0,2 | 1,0 | 0,1 | 0,4 | 0,1 | 0,9 | 0,3 | 0,6 | 0,1 | 0,7 | 0,3 | 0,5 | 0,1 | 0,7 | 0,1 | 0,6 | 0,2 | 0,4 | 0,1 | 0,7 | 0,0 | 0,6 | 0,1 | 0,6 | 0,0 |
| Mcam | 0 | 0,1 | 1,3 | 0,1 | 0,4 | 0,1 | 1,4 | 0,3 | 0,9 | 0,1 | 0,7 | 0,2 | 0,6 | 0,1 | 0,8 | 0,1 | 0,6 | 0,1 | 0,7 | 0,2 | 1,0 | 0,0 | 0,8 | 0,1 | 0,6 | 0,1 |
| Celsr2 | 0 | 0,1 | 1,0 | 0,1 | 0,3 | 0,0 | 0,8 | 0,2 | 0,7 | 0,1 | 0,6 | 0,3 | 0,8 | 0,5 | 0,5 | 0,2 | 0,3 | 0,1 | 0,5 | 0,2 | 0,7 | 0,1 | 0,5 | 0,1 | 0,6 | 0,2 |
| Daam1 | 0 | 0,2 | 1,3 | 0,2 | 0,3 | 0,1 | 1,0 | 0,3 | 0,8 | 0,1 | 0,7 | 0,2 | 0,5 | 0,1 | 0,7 | 0,1 | 0,4 | 0,1 | 0,5 | 0,1 | 0,9 | 0,1 | 0,4 | 0,1 | 0,6 | 0,0 |
| PTK7 | 0 | 0,1 | 1,0 | 0,2 | 0,2 | 0,1 | 1,4 | 0,2 | 1,0 | 0,1 | 0,7 | 0,3 | 0,8 | 0,3 | 0,6 | 0,1 | 0,6 | 0,1 | 0,5 | 0,1 | 0,7 | 0,1 | 0,7 | 0,1 | 0,5 | 0,1 |
| DKK2 | 0 | 0,3 | 1,0 | 0,3 | 1,9 | 0,5 | 2,5 | 1,1 | 1,8 | 0,1 |  | 0,2 | 1,1 | 0,4 | 0,8 | 0,1 | 1,0 | 0,1 | 0,8 | 0,2 | 0,7 | 0,2 | 0,6 | 0,1 | 0,5 | 0,3 |
| DKK3 | 0 | 0,2 | 1,0 | 0,1 | 0,3 | 0,1 | 0,8 | 0,3 | 1,2 | 0,1 | 0,7 | 0,3 | 0,5 | 0,1 | 0,5 | 0,2 | 0,4 | 0,1 | 0,7 | 0,4 | 0,7 | 0,1 | 0,6 | 0,2 | 0,5 | 0,1 |
| DKK4 | 0 | 0,1 | 1,2 | 0,1 | 0,6 | 0,1 | 2,1 | 0,2 | 1,0 | 0,1 | 0,8 | 0,1 | 0,7 | 0,1 | 0,9 | 0,1 | 0,6 | 0,0 | 0,7 | 0,0 | 0,8 | 0,0 | 0,7 | 0,1 | 0,7 | 0,0 |
